# Snapshots of nascent RNA reveal cell- and stimulus-specific responses to acute kidney injury

**DOI:** 10.1172/jci.insight.146374

**Published:** 2022-03-22

**Authors:** Tian Huai Shen, Jacob Stauber, Katherine Xu, Alexandra Jacunski, Neal Paragas, Miriam Callahan, Run Banlengchit, Abraham D. Levitman, Beatriz Desanti De Oliveira, Andrew Beenken, Madeleine S. Grau, Edwin Mathieu, Qingyin Zhang, Yuanji Li, Tejashree Gopal, Nathaniel Askanase, Siddarth Arumugam, Sumit Mohan, Pamela I. Good, Jacob S. Stevens, Fangming Lin, Samuel K. Sia, Chyuan-Sheng Lin, Vivette D’Agati, Krzysztof Kiryluk, Nicholas P. Tatonetti, Jonathan Barasch

**Affiliations:** 1 Department of Medicine, and; 2 Department of Biomedical Informatics, Columbia University, New York, New York, USA.; 3 Department of Medicine, University of Washington, Seattle, Washington, USA.; 4 Department of Biomedical Engineering,; 5 Department of Epidemiology,; 6 Department of Pediatrics, and; 7 Department of Pathology and Cell Biology, Columbia University, New York, New York, USA.

**Keywords:** Nephrology, Chronic kidney disease

## Abstract

The current strategy to detect acute injury of kidney tubular cells relies on changes in serum levels of creatinine. Yet serum creatinine (sCr) is a marker of both functional and pathological processes and does not adequately assay tubular injury. In addition, sCr may require days to reach diagnostic thresholds, yet tubular cells respond with programs of damage and repair within minutes or hours. To detect acute responses to clinically relevant stimuli, we created mice expressing Rosa26-floxed-stop uracil phosphoribosyltransferase (*Uprt*) and inoculated 4-thiouracil (4-TU) to tag nascent RNA at selected time points. Cre-driven 4-TU–tagged RNA was isolated from intact kidneys and demonstrated that volume depletion and ischemia induced different genetic programs in collecting ducts and intercalated cells. Even lineage-related cell types expressed different genes in response to the 2 stressors. TU tagging also demonstrated the transient nature of the responses. Because we placed *Uprt* in the ubiquitously active Rosa26 locus, nascent RNAs from many cell types can be tagged in vivo and their roles interrogated under various conditions. In short, 4-TU labeling identifies stimulus-specific, cell-specific, and time-dependent acute responses that are otherwise difficult to detect with other technologies and are entirely obscured when sCr is the sole metric of kidney damage.

## Graphical abstract

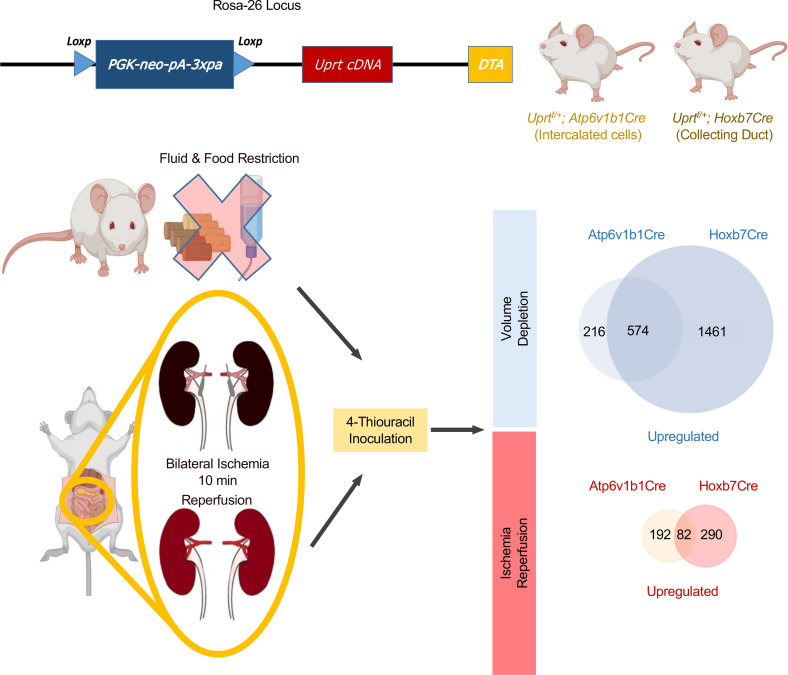

## Introduction

Many different types of stimuli challenge the homeostatic functions of the kidney, and in some cases, damage its cells. While it may be possible to identify many of these stimuli by clinical history, the current clinical metrics of kidney injury do not focus on the presumptive stimulus or on the cellular anatomy of injury. Rather, the current diagnosis of kidney injury measures kidney failure to transfer water and creatinine from the serum to urine, known as “acute kidney injury” (AKI) (1–3).

Serum creatinine (sCr) levels reflect the synthesis of creatinine by muscle cells and its clearance by filtration into the urine — a process described by the mass transfer equation, which yields the glomerular filtration rate. According to this equation, sCr levels reflect the excretory function of the kidney at steady state but not during periods when physiologic functions rapidly decline. As a result, sCr inadequately measures functional loss at the time of patient encounter because it cannot match the kinetic demands of the injury. In fact, sCr may not even achieve minimal criteria for diagnosis (as defined by Acute Kidney Injury Network [AKIN]; Risk, Injury, Failure, Loss of kidney function, and End-stage kidney disease [RIFLE]; or Kidney Disease: Improving Global Outcomes [KDIGO]) (3, 4) when less than half the number of nephrons are injured, a manifestation of “renal reserve” (5–7), nor does elevated sCr unequivocally suggest cellular injury since most patients achieving KDIGO criteria experience resolution almost immediately (33% of cases normalize by 24 hours and ~75% by 72 hours) (8, 9). Hence, it is not surprising that it is difficult to correlate sCr with histologic or molecular findings in humans and mice (10–14). These shortcomings affect real-time decision-making in pressured clinical settings, such as the emergency department (15). Perhaps, a molecular approach to kidney injury might better align with etiologic information and with the patient’s clinical course, particularly when molecular and physiologic markers are considered in combination (16–19).

In order to develop diagnostics that directly measure tubular injury, a complete catalog of cellular and molecular responses to different stimuli is required. For example, an injurious stimulus might affect all kidney cells equally, or instead, it might activate different molecular processes in different cells. It is also possible that some stimuli affect only 1 cell type or only 1 segment of the kidney. Hence, it is not clear whether a stimulus is paired with a specific transcriptional response, whether a final common pathway is activated by different stimuli, or whether the intensity of a stimulus modulates the type or location of the response. The kinetics of the cellular response adds additional complexity since different molecular programs may appear or disappear over time. In sum, a stimulus-response map might contain stimulus-specific, cell-specific, and time-specific readouts that detail the anatomy and evolution of injury.

Here we have adapted a method called thiouracil tagging to obtain snapshots of newly synthesized RNA from a cell type of interest, directly from intact organs. Any number of stimuli can be mapped with this tool, including different types of diseases and different chemical exposures. To determine whether the method can detect stimulus-specific responses, we created models of volume depletion and ischemia/reperfusion injury, 2 common clinical stimuli associated with kidney injury. To determine whether the method can detect cell-specific responses, we examined the collecting duct because it contains neighboring, embryonically related cell types that respond to both stimuli. Its canonical principal cells and intercalated cells derive from a common precursor (the ureteric double-positive cells expressing both principal and intercalated cell markers) and share Notch/Jag1 signaling (20) due to the coexpression of *Tfcp2l1*, suggesting the possibility of coordinated responses to injury. In addition, because principal and intercalated cells are present in limited numbers (only 2%–3% of total kidney cells), the collecting duct provides a test of the method’s sensitivity. Finally, the particularly rapid expression of the biomarker Ngal (*Lcn2*) in response to ischemia but not in response to volume depletion (14, 18, 21–24) suggests that a kinetically sensitive RNA-labeling method may be particularly useful to distinguish stimulus-specific and cell-specific responses in collecting ducts. Thiouracil tagging provides a unique tool to identify time-dependent transcriptomic profiles in vivo.

Our data indicate that injurious stimuli trigger nascent pools of RNA, depicting molecularly unrelated stimulus-specific and cell-specific responses. However, the patterning of gene expression also suggested the possibility that the boundaries between these types of responses can be blurred when “two distinct hits” stimulate the nephron.

## Results

### Human kidney biopsies reveal complex patterns of gene expression.

**Figure 1:**
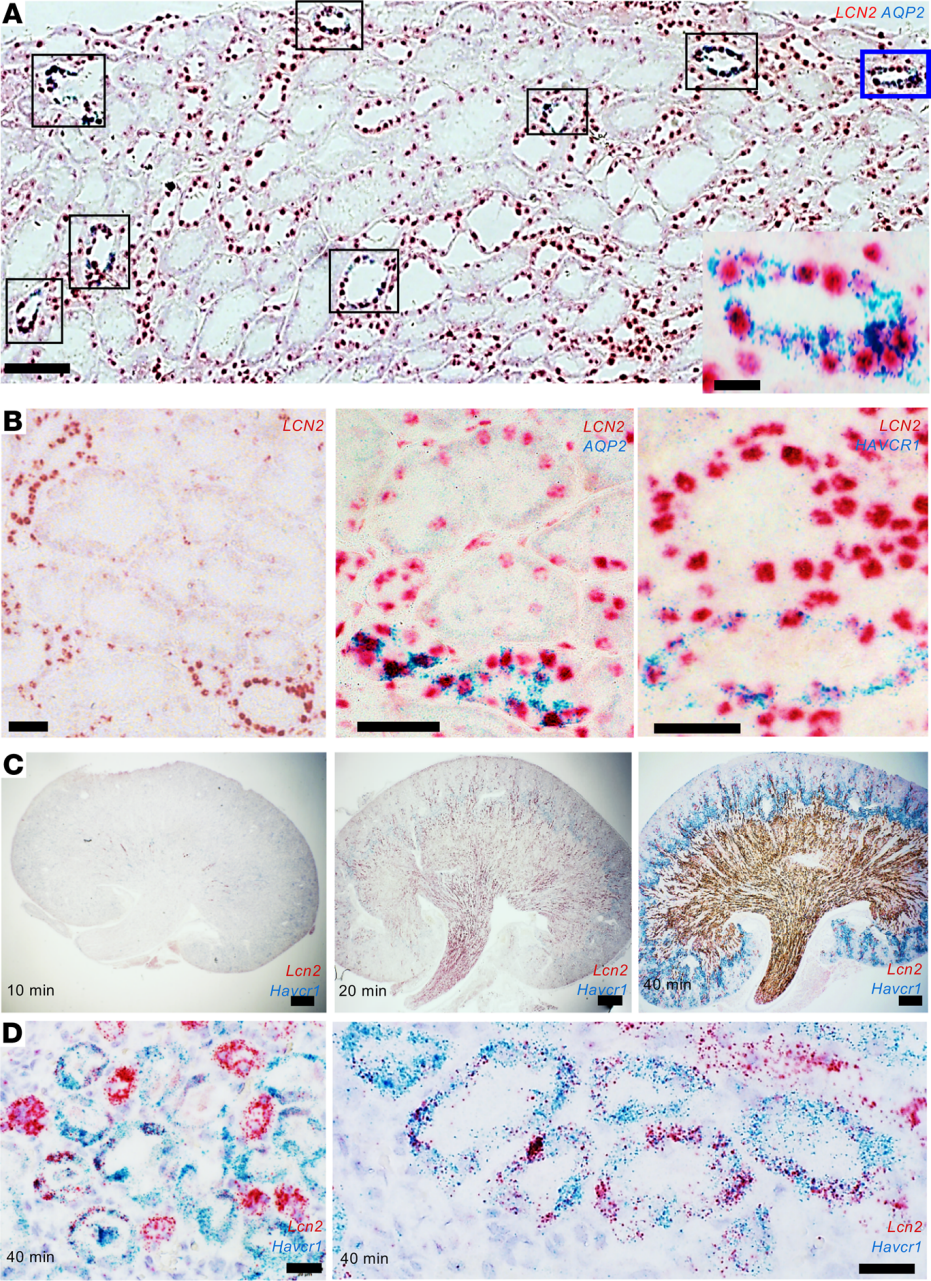
Patterning of gene expression. (**A**) Human kidney biopsy. Mild ATI due to NSAIA. *LCN2* (NGAL) is strongly expressed in *AQP2*^+^ collecting ducts (e.g., boxed tubules) containing both intercalated cells (*AQP2*^–^) and principal cells (*AQP2*^+^) expressing *LCN2* (NGAL). One tubule is outlined and shown in the inset. (**B**) Human kidney biopsies. Left: Ischemic ATI with hepatorenal syndrome. Middle: Nephrectomy — 18 minutes of warm ischemia followed by cold ischemia. Right: Delayed graft function and tacrolimus toxicity. Note *AQP2* (middle) and *HAVCR1* (KIM-1; right) mark collecting ducts and proximal tubules, respectively. Distal nephron segments consistently express *LCN2* (NGAL), but proximal tubule expression is variable. A total of 2 nephrectomies and 5 biopsies were analyzed. (**C**) Cross-clamping the mouse renal pedicle for 10–40 minutes resulted in the progressive broadening of *Lcn2* (Ngal) message throughout the medullary and cortical-medullary zones and *Havcr1* (Kim-1) message in the cortex and outer stripe of the outer medulla. (**D**) Severe ischemia (40 minutes) induced overlapping *Lcn2* (Ngal) and *Havcr1* (Kim-1) expression in the proximal tubule. ISH detected by RNAscope; red = *LCN2*; blue = *AQP2* or *HAVCR1* (KIM-1). Bars **A** = 100 μm; bars **A** (inset) = 10 μm; bars **B** = 50 μm; bars **C** = 500 μm; bars **D** = 20 μm.

To identify the patterning of injury genes, we first examined *LCN2* (NGAL) and *HAVCR1* (KIM-1), 2 established injury biomarker genes. We examined 2 human nephrectomies (~15 minutes warm ischemia) and 5 human biopsies with a range of pathological diagnoses, including rhabdomyolysis, tacrolimus toxicity, acute tubular injury (ATI) possibly secondary to urinary tract infection, and ischemic ATI in the setting of hepatorenal syndrome or in the setting of a nonsteroidal antiinflammatory agent (NSAIA). We found a reproducible pattern of *LCN2* (NGAL) expression in all the kidneys. *LCN2* (NGAL) mRNA was prominently expressed in principal (aquaporin 2–positive, *AQP2*^+^) and intercalated cells (*AQP2*^–^) of the collecting ducts (Figure 1A) and in the presumptive limbs of Henle. In addition, close inspection of different biopsies revealed variable levels of *LCN2* (NGAL) mRNA in cortical proximal tubules *HAVCR1^+^* (KIM-1) ranging from undetectable to intense staining (Figure 1B). The patterning of *LCN2* (NGAL) expression was validated with different chromogens and serial dilution of the probes (Supplemental Figure 1; supplemental material available online with this article; https://doi.org/10.1172/jci.insight.146374DS1). Similar to human kidneys, ischemic mouse kidneys expressed dose-responsive, distinct distributions of *Lcn2* (Ngal; distal nephron) and *Havcr1* (Kim-1; proximal nephron) (Figure 1C) as well as overlapping expression in proximal tubules after prolonged ischemia (Figure 1D) (21). Hence, different stimuli generate both common and variable cell-specific responses.

### Animal model for the isolation of newly synthesized RNA.

The identification of expression patterning beyond a few well-studied genes requires an unbiased method of RNA detection. In addition, a time-sensitive method would be particularly useful given the rapid changes in gene expression after injury (e.g., *Lcn2* [Ngal] transcripts elevate 2–3 hours after a stimulus but regress after 24 hours; refs. 22, 25, 26).

**Figure 2:**
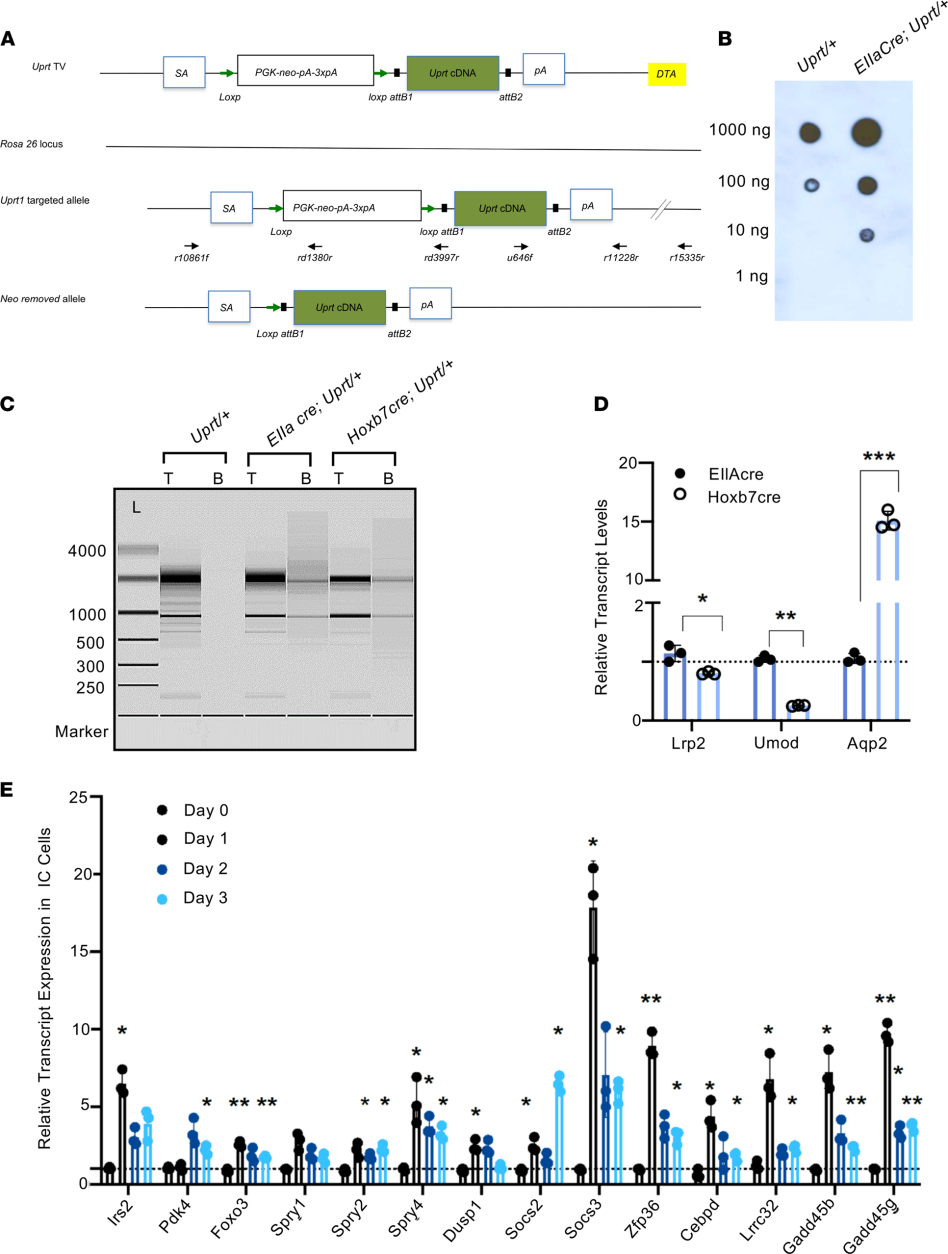
Cell- and time-specific labeling of nascent RNA by UPRT. (**A**) Uprt mice. A knockin vector was created with *Uprt* cDNA flanked by a floxed-stop cassette. *Rosa26^Uprt^* is expressed after Cre-mediated deletion of *PGK-neo-pA-3xpA*. *DTA*, diphtheria toxin A; *SA*, splicing acceptor; *pA*, poly(A) tail; TV, targeting vector. (**B**) Dot blot analysis of biotinylated RNA. Total RNA was extracted from kidneys of 3-month *Rosa^Uprt/+^* and *Rosa^Uprt/+^ EIIaCre* littermates, thio-biotinylated in vitro, and spotted on zeta membranes. The thio-biotinylated RNAs were detected with streptavidin-HRP and visualized with ECL. RNA isolated from *Uprt*-expressing kidneys (*EIIaCre Rosa^Uprt/+^*) was efficiently biotinylated. (**C**) Capture of nascent RNA from Uprt^+^ mice by magnetic beads. A total of 1 ng of total kidney RNA (T) and 0.14 ng of biotinylated RNA were loaded on RNA pico-chips and analyzed by Agilent 2100 Bioanalyzer. RNA size ladder (L) in nt at left. Note that ribosomal RNA was de-enriched in the biotinylated RNA pool. (**D**) Snapshots of nascent RNA. *Aqp2*, a collecting duct marker, was enriched in the *Hoxb7Cre Rosa^Uprt/+^* RNA pool compared with the *EIIaCre Rosa^Uprt/+^* pool, whereas markers of TALH and proximal tubule, *Umod* and *Lrp2*, respectively, were de-enriched. *Actb* was an internal control. Transcript levels in EIIaCre were set to 1. *P < 0.05; **P < 0.01; ***P < 0.001. Data represent mean ± SD. Student’s 2-tailed *t* test (*n* = 3 technical replicates). (**E**) Time-dependent gene expression. *Atp6v1b1Cre Rosa^Uprt/+^* mice (3 months old) were treated with water and food withdrawal for 0, 1, 2, and 3 days. 4-TU was administered and labeled RNA was isolated for reverse transcriptase quantitative PCR (RT-qPCR) after 4 hours. mRNA expression level of day 0 control was set as 1; *Actb* was a reference. Bonferroni-adjusted significance: *P < 0.05, **P < 0.01, ***P < 0.001 compared with day 0 control (2-tailed Student’s *t* test). *n* = 3 technical replicates. Data represent mean ± SD.

To fulfill these criteria, we adapted the method of Cleary and Doe et al. (27, 28). Uracil phosphoribosyltransferase (*Uprt*) was subcloned into the Rosa26 locus by homologous recombination under control of a transcriptional stop cassette, *PGK-neo-pA-3xpA* (Figure 2A). *Rosa^Uprt/+^* mice were bred with 1 of 3 Cre reporter strains to examine cell types of relatively low abundance: Hoxb7Cre induced *Uprt* expression in the collecting duct; Atp6v1b1Cre generated more focused expression in the intercalated and double-positive cells (20) of the collecting duct; and EIIaCre activated *Uprt* throughout the kidney.

To validate the *Rosa^Uprt/+^* construct, we inoculated *EIIaCre Rosa^Uprt/+^* mice with 4-thiouracil (4-TU), an analog that is incorporated into nascent RNA by Uprt but not by wild-type phosphoribosyltransferases. Isolation of 4-TU–tagged RNA was optimized using thio-biotinylation of total RNA extracts from *EIIaCre Rosa^Uprt/+^* kidneys and avidin-bead precipitation (Supplemental Figure 2). Thio-biotinylated mRNA was detected by nitrocellulose blotting with streptavidin-HRP. In contrast, in the absence of Cre, *Rosa^Uprt/+^* mice demonstrated more than 10-fold less labeling (Figure 2B).

The thio-biotinylated RNAs were largely depleted of ribosomal RNA, but they were enriched for Cre-dependent RNA species (Figure 2C). For example, segment-specific labeling with *Hoxb7Cre Rosa^Uprt/+^* enriched collecting duct markers, such as *Aqp2*, and depleted *Umod* (the marker of the thick ascending loop of Henle, TALH) and *Lrp2* (Megalin, the marker of the proximal tubule) from total kidney RNA, prepared in parallel by thio-biotinylation using *EIIaCre Rosa^Uprt/+^*. Hence, the 4-TU protocol distinguished broadly different pools of RNA based on different Cre drivers (Figure 2D).

Next, we validated 2 well-described Cre drivers that target different populations of collecting duct cell types. The drivers were validated using nuclear or membrane-expressing Rosa-Tomato-GFP mice. *Hoxb7Cre Rosa^nT-nG/nT-nG^* generated nuclear GFP^+^ kidney tubules and yielded living GFP^+^, calcein AM^+^ cells upon FACS (Supplemental Figure 3). Compared with unsorted single cells prepared from the whole kidney, *Hoxb7Cre Rosa^nT-nG/nT-nG^* enriched collecting duct principal cell genes and depleted TALH, proximal tubule, and glomerular genes. *Atp6v1b1Cre Rosa^mTmG/mTmG^* labeled a subset of the collecting duct cells enriched in intercalated cell genes but depleted principal cell, TALH, proximal tubule, and glomerular genes (Supplemental Figure 3). *Atp6v1b1Cre Rosa^mTmG/mTmG^* also detected a few scattered interstitial cells in some kidneys. To determine the identity of these cells, we scanned the FACS-isolated cells for macrophage markers *Itgam* (Cd11b), *Emr1*, *Cd68*, *Csf1R*, *Lgals3*, *Itgax*, *Ly6g6d*, *Ly6c1*, *IL4ra*, and *Cd163*. However, most markers were not enriched in the isolated cells, and in fact, *Itgam* is expressed by some kidney tubules (29, 30). *Cd163* was the only macrophage marker variably enriched in RNA pulldowns from *Atp6v1b1Cre* *Rosa^Uprt/+^* mice, but since *Cd163*^+^ macrophages are not abundant in the kidney (31), this minor component may be a different type of interstitial cell. Hence the Cre drivers demonstrate adequate cell specificity.

**Figure 3:**
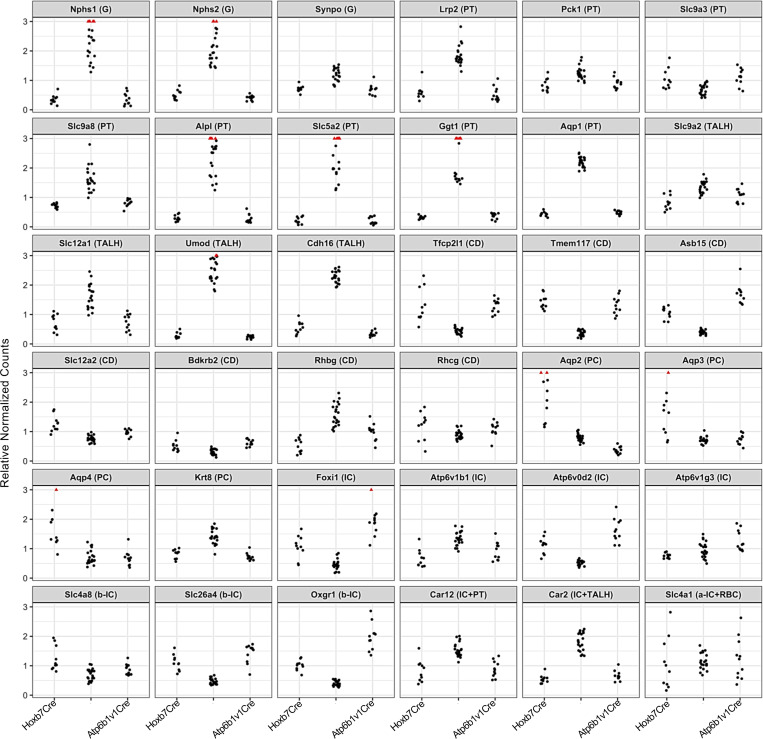
Enrichment of kidney segment markers in nascent RNA. Normalized counts (DESeq2) were plotted for 3 RNA pools (total kidney RNA; *Hoxb7Cre Rosa^Uprt/+^* and *Atp6v1b1Cre Rosa^Uprt/+^* pulldown RNA; respectively, *n* = 10, 21, 11 independent RNA samples). The *y* axis represents the ratio of counts per library size normalized for the average count of each gene across all samples. G, glomerulus; PT, proximal tubule; TALH, thick ascending limb of Henle; CD, collecting duct; PC, principal cells of the collecting duct; IC, intercalated cells of the collecting duct; α-IC, α-intercalated cells, β-IC, β-intercalated cells. Note the de-enrichment of most G, PT, and TALH markers and conversely the enrichment of PC markers by HoxB7Cre (*Aqp* *2*, *3*, *4*), IC markers by Atp6v1b1Cre (*Foxi1*, *Atp6v0d2*, *Atp6v1g3*, *Oxgr1*), and collecting duct markers in both pulldowns (*Rhcg*, *Slc4a8*, *Slc26a4*). Some genes failed to comply with their known status as a canonical marker, suggesting low rates of RNA turnover or incomplete characterization of their distribution, including *Slc9a3* (95). Alternatively, some genes are expressed in multiple segments of the kidney — *Rhbg* (96): connecting segment and CD; *Car12* (carbonic anhydrase 12) (32): PT and CD; *Car2* (carbonic anhydrase 2) (97): TALH and CD; *Slc4a1* (98): RBC and CD — and consequently demonstrated mixed patterns in RNA pulldowns. HoxB7Cre pulldown vs. total RNA: adjusted *P* (padj) < 10^–3^ (range: 3.0 × 10^–3^ to 2.2 × 10^–104^) for all genes except *Rhcg*, *Atp6v1g3*, *Slc4a1* (padj = NS). Atp6v1b1Cre pulldown vs. total RNA: padj < 10^–3^ (range: 7.7 × 10^–3^ to 2.07 × 10^–126^) for all genes except for *Rhcg*, *Slc4a1*, *Atp6v1b1*, *Slc9a2*, *Aqp3*, *Aqp4* (padj = NS).

Using the validated Cre drivers, we tested whether the Uprt protocol could distinguish RNA in the 2 highly related cell populations. We compared thio-biotinylated RNA pools from principal and intercalated cell types by generating *Hoxb7Cre* *Rosa^Uprt/+^* and *Atp6v1b1Cre Rosa^Uprt/+^*, respectively. To identify nascent RNA, we pulsed the mice with 4-TU for 4 hours before isolating the RNA. In dozens of pulldowns of newly synthesized RNA, we found de-enrichment of markers of the glomerulus, proximal tubule, and TALH, and we found enrichment of markers of the collecting duct and its principal and intercalated cells (Figure 3). PC markers (e.g., *Aqp2*, *Aqp3*, *Aqp4*) were enriched in the Hoxb7Cre thio-biotin pool, and intercalated cell markers (e.g., *Foxi1*, *Atp6v1b1*, *Atp6v0d2*, *Slc26a4*) were enriched in the Atp6v1b1Cre thio-biotin pool (Supplemental Tables 1 and 2 — baseline transcriptomes). Many of the same genes were identified independently in *nT-nG/mTmG* FACS-purified collecting duct and intercalated cells, showing that Uprt identified transcripts in anatomically defined cells (Supplemental Figure 3). The numerical enrichment score for a given RNA species depended on the overlap of the expression domain of the gene and the Cre driver. For example, intercalated cell genes *Car2* and *Car12* were expressed at multiple sites in the kidney and consequently were not enriched in the Atp6v1b1Cre pulldowns (32, 33). Hence, Cre-driven Uprt labeling allows isolation of cell type–specific RNA. Even Cre drivers with overlapping domains, such as Hoxb7Cre and Atp6v1b1Cre, can still demonstrate cell-specific enrichment.

### Stimulus-specific gene expression.

**Figure 4:**
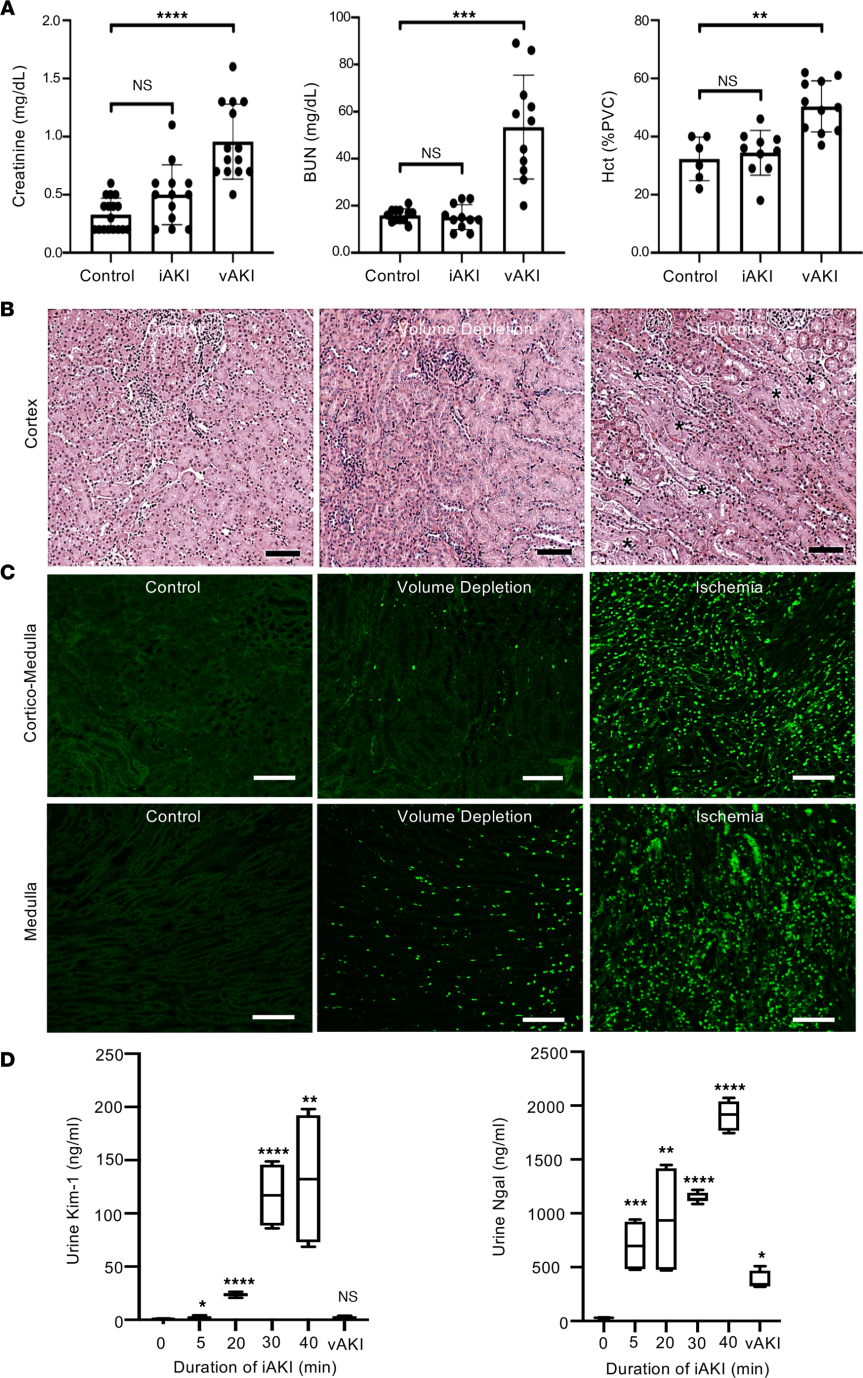
Models of different forms of azotemia. (**A**) Ischemic injury of the kidney elevated sCr at the 24-hour time point (*P* = NS; control *n* = 16, ischemia *n* = 13) but had no effect on BUN (*P* = NS) or hematocrit (HCT; *P* = NS). Volume depletion (volume depletion) elevated sCr 3-fold (*P* < 10^–5^; volume depletion *n* = 14), BUN 3-fold (*P* < 0.001; control *n* = 11, volume depletion *n* = 11), and HCT 1.5-fold (*P* < 0.01; control *n* = 6, volume depletion *n* = 11). Ischemia differed from volume depletion in all 3 parameters. Significance was determined by Student’s 2-tailed *t* test. *P* values were Bonferroni corrected. Data represent mean ± SD. Bars = 100 μm. PVC, packed volume of cells. (**B**) Renal artery ischemia generated focal coagulative necrosis of tubules (*) while volume depletion failed to demonstrate evidence of extensive kidney damage. H&E stain. Bars = 100 μm. (**C**) Renal artery ischemia generated TUNEL^+^ cell death (green) throughout the cortico-medullary junction and medulla, whereas volume depletion had little effect, except for a few scattered cells in the medulla. TUNEL was assayed by Click-iT Plus TUNEL kit (Invitrogen). Bars = 100 μm. (**D**) Urinary tubular injury markers Kim-1 and Ngal were induced by ischemic injury in a dose-dependent fashion but demonstrated limited responses to prolonged volume depletion (3 days) despite higher sCr levels. Urine Ngal and Kim-1 were quantified by ELISA (mouse Ngal: R&D Systems MLCN20; mouse Kim-1: R&D Systems MKM100) (*n* = 4) and compared with normal control. (**A** and **D**) *P < 0.05; **P < 0.01; ***P < 0.001; ****P < 0.00001 (2-tailed Student’s *t* test). *P* values were Bonferroni corrected. The box plots depict the minimum and maximum values (whiskers), the upper and lower quartiles, and the median. The length of the box represents the interquartile range.

Current criteria for AKI suggest that any elevation of sCr meeting RIFLE, AKIN, or KDIGO criteria marks an injury to kidney cells and consequently should correlate with tubular injury genes. However, if the type of stimulus was also important, then distinct genes should be induced by different stimuli. To evaluate whether the Uprt protocol can distinguish stimulus-dependent responses, we employed 2 extensively characterized models with elevated sCr (9). The arterial-venous ischemic model employed bilateral renal pedicle clamping for 10 minutes followed 24 hours later by 4-TU RNA labeling. The volume-depleted model employed restriction of water and food for 72 hours, followed by 4-TU RNA labeling. RNA harvest was timed so that both models demonstrated elevated levels of sCr (doubling in the ischemic model and rising 3-fold in the volume-depleted model). The volume-depleted model also resulted in a greater rise in blood urea nitrogen (BUN) and hemoconcentration, typical findings of volume depletion in both mice and humans (Figure 4A). Nonetheless, even 3 days of volume depletion demonstrated limited histologic evidence of tubular disruption (Figure 4B), limited TUNEL staining (Figure 4C), and limited induction of Ngal and Kim-1, urinary biomarkers of tubular injury (Figure 4D). In contrast, focal coagulative necrosis and prominent TUNEL staining were found in both cortical and medullary tubules subsequent to 10 minutes of ischemia. Increasing doses of ischemia (0, 5, 20, 30, 40 minutes of arterial clamping) increased urine Ngal and Kim-1 as much as 70- and 130-fold, respectively. These data are consistent with both the Ngal-reporter mouse (22) and human cohorts in emergency departments (23, 34) and in other settings (14, 18, 24).

**Figure 5:**
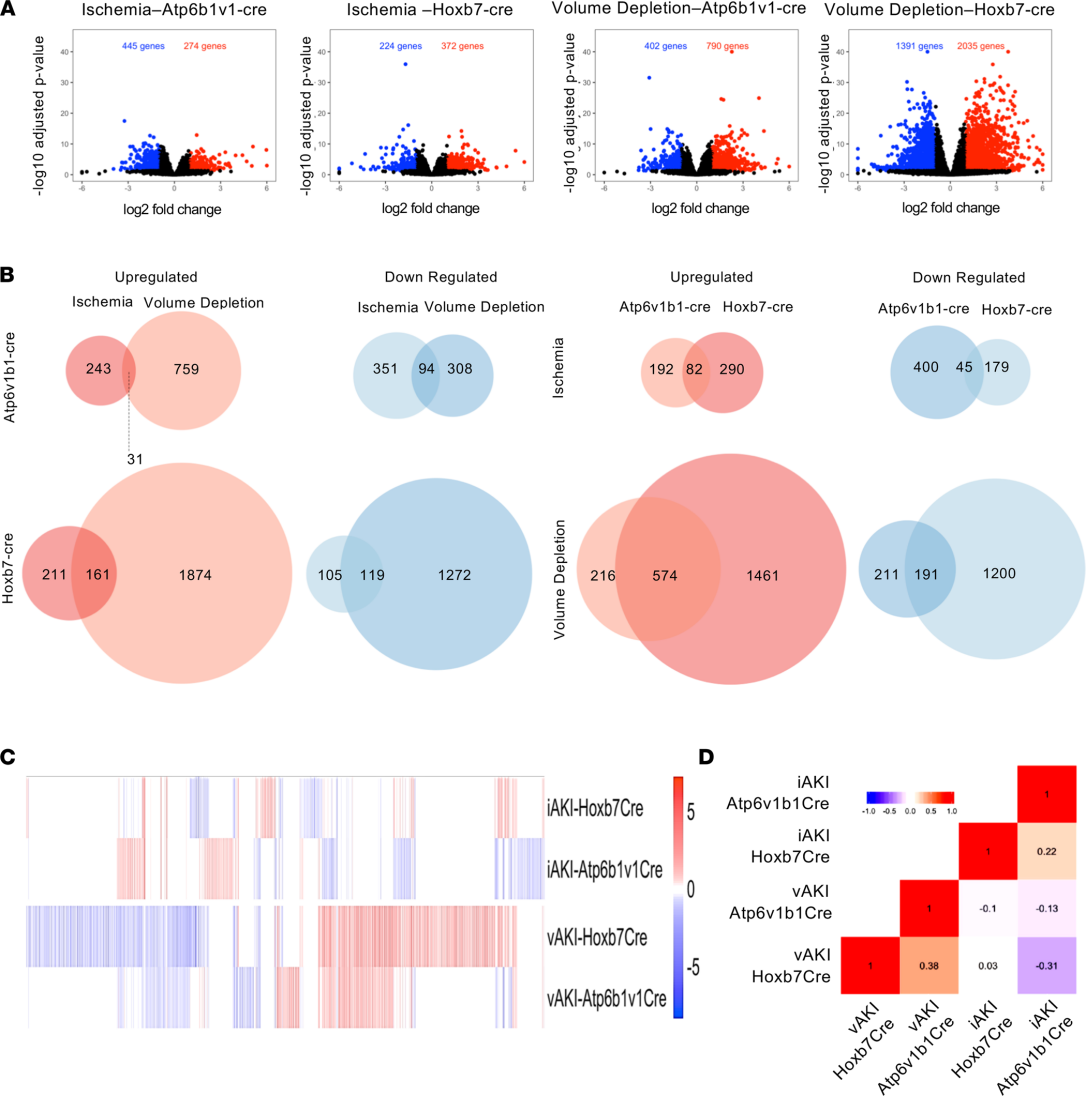
Response to AKI differs according to stimulus and cell type. (**A**) Comparison of differential gene expression. Hoxb7-Cre = collecting duct; Atp6v1b1-Cre = intercalated cells and their derivatives. Volcano plot shows log_2_ fold change for all DEGs with cutoff –1, +1 and padj < 0.05 in each RNA pool. (**B**) The Venn diagrams represent significantly upregulated and downregulated DEGs (log_2_ fold ≥ 1; padj < 0.05). The numbers in the Venn diagrams represent the number of DEGs shown in **A**. Note the differential responses to ischemia and to volume depletion (stimulus-specific responses) in the collecting duct. Also note that the 2 Cre drivers enrich for different sets of genes despite overlapping RNA pools (cell-specific responses). (**C**) Heatmap of all DEGs according to stimulus and cell type (padj < 0.05). (**D**) Pearson correlation matrix of all DEGs according to stimulus and cell type. Numbers represent correlation coefficients (*P* < 10^–4^). *n* = 7, volume depleted; *n* = 7, ischemia; control, *n* = 8.

The distinctive responses of the kidney to volume depletion and ischemia provided a useful test of the Uprt method to detect stimulus-dependent responses. Hierarchical clustering of *z* score–transformed, normalized counts (transcripts per million [TPM]; padj < 0.05 and log_2_ fold change ≥ 1) of nascent RNA demonstrated differentially expressed genes (DEGs). Both male and female mice distinguished the 2 stimuli, but, because variance of gene expression was greater in female than in male mice, we present the latter in tabular and graphical form (Supplemental Figure 4). As shown in Figure 5 (see also Supplemental Tables 3 and 4), pulldown of nascent RNA from ischemic *Hoxb7Cre Rosa^Uprt/+^* kidneys generated 596 DEGs (372 upregulated; 224 downregulated), whereas volume depletion generated 3426 DEGs (2035 upregulated and 1391 downregulated) compared with the control pulldown. A direct comparison of ischemia and volume depletion revealed 3462 genes unique to either stimulus. A Venn diagram (padj < 0.05 and log_2_ fold change ≥ 1) illustrates that only a few genes were shared by both models (Figure 5, B and C). Further inspection of Hoxb7Cre RNA pulldowns showed 24 DEGs that were regulated in opposing directions by ischemia and volume depletion (Supplemental Table 5; padj < 0.05). These data demonstrate that different stimuli induce unique transcripts even in a single segment of the kidney. The unique transcripts were detected in the pool of newly synthesized RNA by the Uprt technique.

The Hoxb7Cre driver labels many cell types in the collecting duct. To determine whether stimulus-dependent responses can be reproduced in a subset of collecting duct cells, we examined responses to ischemia and volume depletion using the Atp6v1b1Cre driver (padj < 0.05 and log_2_ fold change ≥ 1). We found that ischemia generated 719 DEGs (274 upregulated; 445 downregulated) while volume depletion generated 1192 DEGs (790 upregulated and 402 downregulated) (Figure 5 and Supplemental Tables 6 and 7). A direct comparison of ischemia and volume depletion revealed 1661 unique DEGs that were uniquely expressed in either ischemia or volume depletion. When depicted in a Venn diagram (padj < 0.05 and log_2_ fold change ≥ 1) we found that there were few genes in common between ischemia and volume depletion (125 genes). Further inspection of Atp6v1b1Cre RNA pulldowns showed 41 DEGs that were regulated in opposing directions (Supplemental Table 8; *P* < 0.05). Hence, both Cre drivers were able to demonstrate stimulus-dependent, nascent DEGs.

### Cell-specific responses to stimuli.

The discovery that biomarkers of kidney injury can derive from different segments of the nephron suggested that injurious stimuli can induce cell-specific responses (Figure 1). To determine whether segment-specific expression is a general principle reproduced in unbiased screens, we used the Uprt technique. We directly compared Hoxb7Cre and Atp6v1b1Cre tagged thiouracil-RNA (Figure 5; padj < 0.05 and log_2_ fold change ≥ 1) to determine whether responses to a single stimulus could be resolved in related adjacent cell types. Out of all the unique ischemia DEGs (1180 DEGs), only 10.9% (127 DEGs) were modulated in both Hoxb7Cre and Atp6v1b1Cre pools; 0.7% (8 DEGs) responded oppositely in the 2 pools (Supplemental Table 9). Consequently, 89% (1045 DEGs) of the genes were uniquely regulated in 1 of 2 pulldowns. Out of all the volume depletion DEGs (3842 DEGs), 19.9% (765 DEGs) were modulated in both Hoxb7Cre and Atp6v1b1Cre pools, and 0.28% (11 DEGs) responded oppositely in the 2 pools (Supplemental Table 10). Consequently, 79.8% (3066 DEGs) of genes were differentially regulated in 1 of the 2 pulldowns.

To determine whether the Uprt method can detect cell-specific responses outside the collecting duct, we created *Lrp2^CreERT2/+^ Rosa^Uprt/+^* mice (Supplemental Figure 5). In a preliminary analysis, renal ischemia/reperfusion injury resulted in 1666 upregulated and 1538 downregulated differentially expressed nascent transcripts (padj < 0.05). Baseline tubular genes *Slc5a12*, *Gatm*, *Slc34a1*, *Alpl* (S1 segment); *Galnt11*, *Lrp2*, *Slc5a8* (S2 segment); and *Atp11a*, *Slc6a6*, *Slc22a14* (S3 segment) were downregulated, while nascent transcripts associated with injury were prominently upregulated, such as *Spp1*, *Havcr1*, *Serpina10*, *Top2a*, *S100a6*, *Il34*, *Cxcl1*, *Vcam1*, *Lcn2*, and *Krt20* (9). Thirty percent of the modulated nascent transcripts were present in single-cell RNA sequencing of proximal tubule clusters of mouse kidneys subjected to a similar protocol of ischemic injury (21). In addition, only 21% of modulated genes were found in the ischemic *Hoxb7Cre Rosa^Uprt/+^* RNA pulldowns. These data authenticate the use of Uprt-nascent RNA technology to detect different types of responses by different types of epithelial cells.

Taken together, the data indicate that despite the fact that sCr is elevated in all of our models, the stimulus and its target cell determine the transcriptomic responses (Figure 5, B and C). The responses to ischemia and volume depletion were distinct, and the responses of the different cell types were divergent particularly when stimulated with ischemia. It is particularly notable that the Uprt method identified these disparate responses despite related cell identities, overlapping Cre driver domains, and limited representation of cell types.

### Pathway analysis of differentially expressed genes.

**Figure 6:**
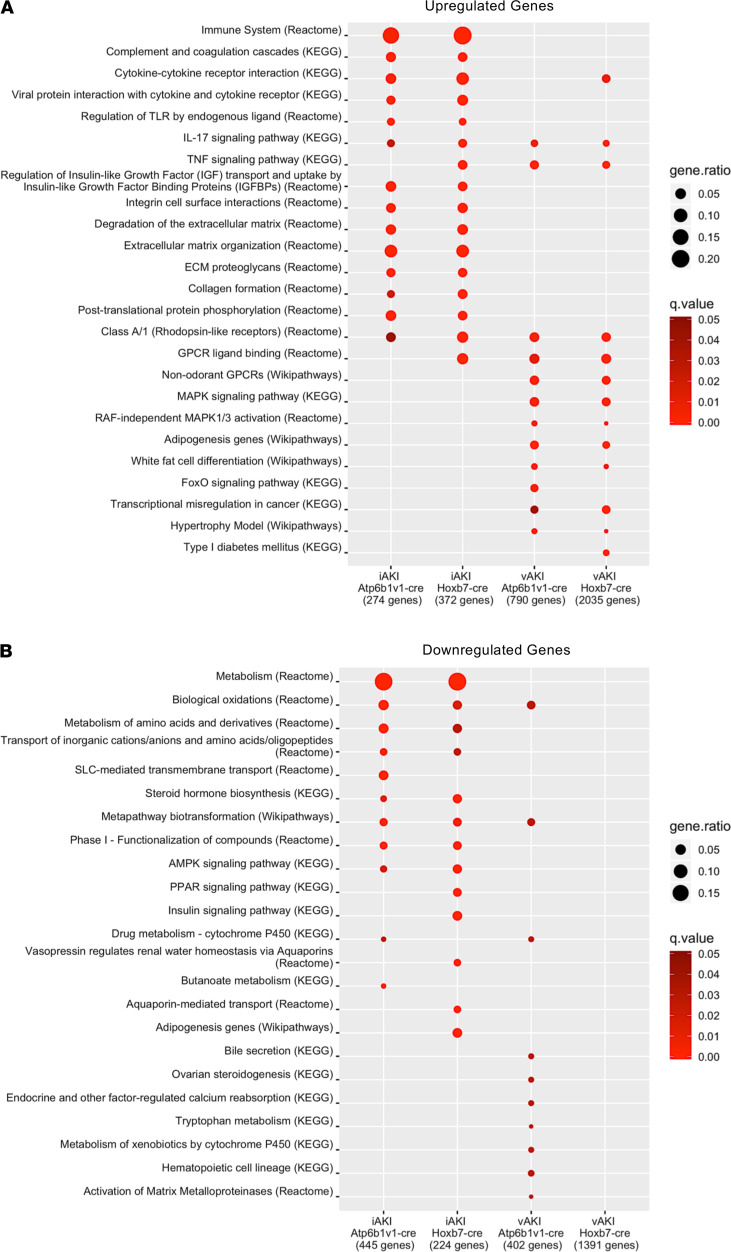
Pathway overrepresentation analysis. Enrichment of pathways in (**A**) significant upregulated DEGs (padj < 0.05; log_2_ fold change ≥ 1) and (**B**) significant downregulated DEGs (padj < 0.05; log_2_ fold change ≤ 1) from each RNA pool. Red color represents –log_10_ *q* value restricted to *q* < 0.05. Note the relatedness of pathways uniquely activated by ischemia or by volume depletion.

The relatedness of gene sets was further tested by overrepresentation analysis. We found that ischemia and volume depletion highlighted unique pathways. In the case of ischemia, reproducible pathways included immune regulation (e.g., immune system, cytokine signaling, TLR signaling), complement cascades, and matrix degradation and collagen turnover (integrin signaling, degradation of matrix, collagen formation). In contrast, in the case of volume depletion, Uprt labeling of nascent RNA highlighted metabolic regulators (FoxO signaling, adipogenesis, and white fat cell differentiation). Many genes activated by volume depletion were in fact inhibitors of inflammatory pathways (Figure 2E and Figure 6).

In sum, RNA pulldowns from the intact kidney showed that ischemia generated immune and clotting reactions and matrix turnover in the collecting ducts, data echoed in the proximal tubule by the enrichment of cytokine and immune signaling. In contrast, volume depletion reset metabolic parameters and produced inhibitors of inflammation. The reciprocal nature of the data sets suggests that ischemic changes in sCr are distinct from volume-induced changes in sCr.

### Snapshots of RNA.

Given that 4-TU labels only RNA undergoing synthesis during the pulse of 4-TU (a 4-hour period), the Uprt technique can provide snapshots of gene expression in response to a stimulus. Since the collecting duct is sensitive to volume depletion, we restricted access to food and water for variable lengths of time (24, 48, or 72 hours). Utilizing preliminary information from unbiased Uprt pulldowns (Supplemental Tables 4 and 7), we selected a series of volume-activated genes and quantified their expression by 4-TU labeling of the kidney at 24, 48, and 72 hours followed by thio-biotinylation, RNA isolation, and RT-qPCR. Most commonly, genetic activation peaked 1 day after the initiation of dietary restriction, but then decayed despite continued restriction (Figure 2E). Hence, Uprt RNA pulldown can be used to identify time-sensitive changes in the transcriptome.

To further test the value of Uprt, we applied the same protocol of volume depletion to both *Atp6v1b1Cre Rosa^Uprt/+^* mice and *Atp6v1b1Cre Rosa^nT-nG/nT-nG^* mice. However, rather than extract nascent intercalated cell RNA directly from the intact *Atp6v1b1Cre Rosa^Uprt/+^* expressing kidneys, intercalated cells from *Atp6v1b1Cre Rosa^nT-nG/nT-nG^* mice were first isolated by kidney dissociation and FACS prior to total RNA extraction. We compared the same genes in the 2 preparations by RT-PCR (Figure 2E and Supplemental Figure 6). While the nascent Uprt RNA pool demonstrated a discrete spike in gene expression on day 1 of volume depletion (e.g., *Irs2*, *Foxo3*, *Dusp1*, *Socs3*, *Zfp36*, *Cebpd*, *Gadd45b*), it was blunted or not apparent at all in RNA isolated from sorted cells. The data suggest that Uprt labeling enhances detection of initial responses that may be less evident in the steady-state RNA pool.

## Discussion

### The Uprt method.

We have adapted the Uprt method developed by Gay et al. (27, 28) to identify the transcriptional responses of kidney cells. Uprt RNA tagging is a particularly powerful tool for the study of acute kidney diseases because it identifies dynamic changes in gene expression by labeling nascent RNA in a brief time interval using a short pulse of 4-TU. In addition, because we subcloned the Uprt enzyme into the Rosa26 locus, *Uprt* can be expressed in any cell of the mouse. Targeted expression is then achieved by removal of the transcriptional stop cassette *PGK-neo-pA-3xpA* by a cell-specific Cre driver. Consequently, the Uprt method permits spatial characterization of gene expression — even in neighboring cells — such as principal and intercalated cells. In addition, this method is sensitive enough to allow for purification of RNA transcribed in rare or dispersed mammalian cell types, such as intercalated cells. Because the technique identifies only actively transcribed RNA, it enhances the identification of stimulus-induced transcripts, providing a useful method to analyze gene expression in tissue injury, which can be limited or patchy in distribution.

It is important to note that the Uprt method does not require overexpression of an endogenous protein that could have deleterious effects. It has no effect on mouse viability and a negligible effect on gene expression of the Uprt construct (27, 28). Furthermore, the technique allows transcriptional labeling of specific cells within intact renal tissues, thus preserving cell interactions and organ physiology without designing freezing protocols, RNA preservatives, or cell dissociation and isolation technologies developed for single-cell assays, nor is there a need for kidney dissection, tubule isolation, or FACS purification to enrich for a specific cell type. A single pulldown from a single kidney is sufficient to generate adequate amounts of RNA for RNA sequencing, even from a minor cell population, and hence there is no need to pool separate batches. In sum, in contrast to dissociation-based methods, where the concern is that the process of purification may trigger a number of changes in gene expression and cause cell stress and death (35, 36), Uprt tagging avoids these additional injuries. As a result, the detection of gene expression is more likely to reflect physiologic responses to stimuli, rather than changes due to the processing of tissues. The strengths and weaknesses of different technologies of identifying cell-specific transcripts are compared in Supplemental Figure 7.

Uprt tagging is complementary to other RNA purification methods such as translating ribosome affinity purification (TRAP) (35). TRAP measures total RNA species stable enough to undergo translation, while Uprt measures snapshots of kinetically active transcription. In addition, whereas TRAP can only label mRNA, Uprt tagging has the potential to purify time-resolved mRNA, noncoding RNA, miRNA, and rRNA (27, 28). Nonetheless, despite these and other technical differences between models, approximately 50% of Hoxb7Cre-driven, Uprt-labeled transcripts induced by ischemia were also found in the Hoxb7Cre-driven Ribotrap gene set (*P* overlap = 0.008) (25). The overlap was specific because no other condition (such as volume stimulation of the kidney) measured by Uprt labeling overlapped with the Ribotrap ischemic set (*P* overlap = NS). The comparison also confirmed the expression of a number of genes known to be associated with ischemic collecting ducts, including *Lcn2* (Ngal), *Timp1*, and *Clu* (clusterin) and revealed unexpected modulation of *Alb* (albumin; typically a serum protein), *Havcr1* (Kim-1) and *Krt20* (both typically associated with the ischemic proximal tubule), and *Lif* and *Crlf* (which can drive tubulogenesis) transcripts.

The Uprt-labeled RNA pools also overlapped with our published laser capture microdissection (LCM) RNA-Seq of large kidney regions subjected to ischemic and volume-depleted conditions (9). By surveying Kyoto Encyclopedia of Genes and Genomes (KEGG), Reactome, and WikiPathways, we found that 50% of pathways were identical in the 2 RNA-Seq databases. In short, Ribotrap and LCM provided complementary data to Uprt snapshots of nascent transcription. While these data supported our analyses, they also demonstrated that the nascent RNA pool differed from the steady-state RNA pool, implying a higher sensitivity to detect early and acute gene expression (Figure 2 and Supplemental Figure 6).

While the Cre-driven tagging of actively transcribed RNA allowed us to characterize the immediate transcriptional response to noxious stimuli in vivo, it also implied certain limitations. First, the Uprt method, by definition, may overrepresent uracil-rich RNA, and this might affect studies requiring absolute quantification and comparison between different genes. These limitations were not problematic here since our focus was on relative changes in the expression of each gene individually, though this consideration may influence the use of pathway and other types of data analyses where the relative weighting of multiple genes is relevant. Second, and most important, the method is not representative of the transcriptome since it enriches for nascent transcripts only and therefore may not fully map onto steady-state databases, such as TRAP or single-cell analyses. For example, if a transcript demonstrates rapid turnover, then it could be highly represented at some point after a stimulus in a Uprt RNA-Seq pulldown but potentially not in steady-state RNA-Seq if the transcript is also rapidly degraded. In other words, it is conceivable that labeling nascent transcripts may represent higher RNA turnover rather than higher levels of translatable RNA. In this light, it is essential to account for baseline differences in expression kinetics with control mice by performing identical pulldowns at the same time as the experimental model. Yet, even then, one must still consider that expression kinetics is likely to vary between different genes, potentially again limiting gene-to-gene comparisons. We suggest that once snapshots of candidate transcripts are identified by serial Uprt assays, their abundance at time points of interest are further analyzed by PCR or other techniques starting with bulk or translatable RNA. In this regard, the Uprt method may be complementary to the TRAP method.

A second set of limitations of the Uprt method is the need to fully characterize the Cre driver. The resolution of Uprt data is determined by the boundaries of Cre expression, and in most tissues, it seems likely that an average profile of similar cells will be obtained. For example, we previously identified a double-positive subset of intercalated cells (Atp6v1b1^+^Aqp2^+^) using lineage tracing, gene knockout, and immunocytochemistry, while other authors identified similar “transitional cells” using single-cell technology (20, 36, 37). In contrast to the single-cell approach, the Uprt method is less likely to distinguish double-positive transitional cells from definitive intercalated cells, unless a specific double-positive transitional cell promoter is identified and amenable to drive Cre. Thus, Uprt and single-cell analyses provide different types of data resolution, namely enhanced kinetic information and enhanced subsegment spatial information, respectively. Indeed, comparison of these technologies is useful because it seems that rapid turnover of RNA in untreated kidneys best overlaps with the double-positive subset of intercalated cells (Xu, Kiryluk, Sims et al., unpublished observations).

Despite limitations imposed by the Cre driver, the Uprt method is an important method to detect differentially timed responses. For example, in the well-characterized response of macrophages to lipopolysaccharide (LPS), response genes are grouped into primary and secondary cohorts (38–40). Primary response genes are preloaded with polymerase II, which constitutively produces low levels of full-length unspliced transcripts. Transcription is regulated at postinitiation steps, and within 1 hour of exposure to LPS, productive elongation generates mature, protein-coding transcripts (41). In contrast, induction of secondary response genes is delayed, since they are regulated by a mechanism that requires new protein synthesis and chromatin remodeling (42). The Uprt method may be very well suited for a kinetic analysis of this pathway, since inoculation with 4-TU can be given at any time after application of LPS; therefore, labeling immediately after injury can be compared with labeling at a later time point to compare primary response genes with secondary response genes.

Since Uprt is permanently activated in the initial cell type by a specific Cre driver, and in all of its descendants, the method might identify changes in cell identity if pulses of 4-TU are administered at different times after a stimulus. If an injured cell does not revert to baseline, but rather expresses genes inappropriate for the initial cell type defined by the Cre driver, then the labeled RNA cohort would change from a Cre-appropriate gene set into a Cre-inappropriate gene set. These unexpected genes may be involved with repair or the redifferentiation of the cell, for example during epithelial-to-mesenchymal transition.

### Stimulus-specific and cell-specific responses.

The Cre-driven Uprt method describes a kinetic and spatially restricted approach to non-steady-state cellular responses. Given that acute injury of any tissue represents a non-steady-state condition, we applied the Uprt method to examine snapshots of RNA to identify acutely regulated transcripts.

We found that the acute response of the kidney was stimulus specific, cell specific, and time dependent, which could not be defined by the level of sCr alone. More than 30 years ago, Zager et al. demonstrated that measurements of tubular cell energetics were preserved in models of volume depletion but not in acute ischemic diseases of the kidney (40). More recently, work with mouse models (9, 41) provided further evidence for stimulus-specific transcriptomic responses. For example, we found that while both ischemic and volume-depleted collecting ducts expressed a variety of inflammatory molecules, it was notable that the volume-depleted model expressed many types of cytokine and inflammatory inhibitors as well as cell survival molecules (Figure 2). These included JAK/STAT inhibitors (*Cish*, *Socs2*, *Socs3*; refs. 42–46), MAPK inhibitors (*Dusp1*, *Spry1*,*2*,*4*; refs. 47–52), transcriptional inhibitors of inflammation (*Foxo3*; refs. 53–55), cell cycle arrest and survival under stress regulators (*Gadd45g*, *Gadd45b*; refs. 56–60), additional apoptotic suppressors (*Irs*, *Nfil3*; refs. 61–63), metabolic regulators of carbon metabolism and energy generation (*Pdk4*, *Nmrk1*, *Cebpd*; refs. 64–68), and most interestingly, *Zfp36*, an RNA-binding protein implicated in the destruction of cytokine transcripts containing AU-rich elements (69, 70). The robust upregulation of these transcripts suggested a form of adaptation and cellular protection present in the volume-depleted collecting duct but not in the ischemic tubule.

The identification of unique cell- and stimulus-dependent transcriptional responses will reveal secreted urine and plasma proteins that may provide noninvasive means of detecting rapidly evolving cellular damage. We have previously identified several secreted proteins in the urine and plasma as markers of cellular injury (71, 72). We found that similar to Yuen et al. (41) and Xu et al. (9), transcript levels of secreted proteins distinguished ischemic and volume-depleted states (Supplemental Table 11; *q* < 0.01). Most notably, we found a number of secreted protein-coding genes that were activated by ischemia and detected with both Atp6v1b1Cre and Hoxb7Cre but that were not appreciably activated by volume depletion (*Lcn2*, *Mmp7*, *Mmp8*, *Cxcl5*, *Timp1*, *Il34*). Conversely, we found secreted protein-coding genes that were activated by volume depletion, but they were not appreciably activated or were inhibited by ischemia (*Wnt11*, *Wnt8b*). Hence, not only pathway analyses, but also distinct profiles of secreted proteins, confirmed the intrinsic differences between ischemia and volume depletion despite reduced renal excretory capacity in both cases. Therefore, the analysis of secreted proteins identified by Uprt may provide diagnostic biomarkers that differentiate tubular injury and volume depletion. Consistent with these findings, we previously found that serum NGAL also contributes to urinary NGAL as a result of kidney injury, but not volume depletion (73, 74). However, further investigation of serum NGAL is required (22, 74, 75) because proteinuria and NGAL fail to correlate reproducibly in different diseases (34, 76), human (aggregates) and mouse (monomer) NGAL have biochemical and structural differences (74, 76), and the transplantation of Ngal-KO kidneys into WT mice suppressed urinary Ngal (22), while WT kidneys transplanted into Ngal-KO mice generated serum Ngal. These types of analyses should be generally applied to all biomarker candidates.

### “Two-hit” hypothesis.

While genetic and molecular evidence point to distinct pathological entities, clinically, volume depletion and ischemia are aggregated into the single diagnostic group called “AKI.” Although this may be a consequence of the problems of diagnostic criteria based solely on sCr and urine output, it also leads to the question of whether volume-induced elevation of sCr is simply an attenuated form of ischemia-induced changes. In other words, because clinical experience suggests that volume depletion that is severe enough or prolonged enough (e.g., hypovolemic or cardiogenic shock) may lead to structural tubular injury (formerly known as acute tubular necrosis or ATI), the question arises as to whether outside of these catastrophic settings, the 2 states represent a continuous spectrum, or independent states of excretory dysfunction (77). We favor the latter hypothesis because our mouse models were subjected to volume depletion as severe and prolonged as possible and elevated sCr to a level greater than the ischemic set, and yet volume and ischemic transcriptional profiles were still distinct. Furthermore, we found that volume-stimulated genes normalized within 24 hours of volume repletion (9), suggesting that these genes constitute an appropriate homeostatic response to extracellular fluid volume depletion without tubular cell damage. Taken together, our findings argue against a “spectrum of injury” model, but rather suggest that the progression from volume depletion to ischemia may require a “second hit.”

The notion of a “two-hit model” is consistent with results of animal studies as well as with well-known clinical phenomena. In healthy animals, for instance, suprapharmacologic doses of gentamicin are needed to cause tubular necrosis. When combined with additional insults, such as volume depletion, however, clinically relevant gentamicin doses are sufficient to cause kidney damage (78). Myoglobin nephrotoxicity follows a similar pattern: its toxicity is enhanced by volume depletion or prostaglandin synthesis inhibition (79). Similar phenomena have been recognized in clinical practice. It is well known, for example, that intravascular volume depletion is a risk factor for nephrotoxicity of chemotherapeutic agents (80), precipitation of light chains (81), as well as numerous other agents such as NSAIAs, aminoglycosides, and amphotericin B (82). The recognition of volume depletion as a predisposing factor for radiological contrast-induced nephropathy has led to the study of extracellular volume expansion as a successful strategy to minimize risk (83) and eventually shaped practice guideline recommendations (84). Furthermore, it is interesting to note that tubular injury is an unusual complication of intravascular volume depletion states such as heart failure (85) or cirrhosis in the absence of other secondary causes of injury such as aminoglycoside therapy or hypotension secondary to blood loss (86). All of these examples suggest that the classical “two-hit” model might provide a conceptual model that is closer to clinical phenomena than a continuum or “spectrum of injury” model.

As a preliminary exercise to test the relevance of the distinction between ischemic and volume-depleted models, we repeated the volume depletion protocol in *Zfp36*-knockout mice. *Zfp36^–/–^* mice (Supplemental Figure 8) have chronic inflammatory disease marked by elevated interleukins (87, 88) but without overt tubular damage (based on urinary biomarkers, immunocytochemistry for Kim-1, and normal sCr levels). *Zfp36* was also markedly elevated in the volume-depleted model (Figure 2E), suggesting that it may have a role in reducing inflammatory responses. However, volume depletion in *Zfp36^–/–^* mice (~18% loss in body weight) not only demonstrated the clinical characteristics of volume depletion (elevated BUN and plasma sodium), along with a potential marker of volume depletion, high–molecular weight *Pappa2* (9) (Supplemental Figure 8B), but also activated classical markers of ischemic tubular damage, which were not expressed in wild-type volume-depleted mice or in the *Zfp36^–/–^* controls. These included urinary Ngal and Kim-1 immunostaining (Supplemental Figure 8, C and D; uncut gel, Supplemental Figure 9). These preliminary data reinforce the notion that elevations of sCr represent independent stable disease entities and that a “second hit” changes the transcriptional output. They are consistent with many types of clinical experiences that suggest a synergistic effect between different types of stimuli. This reasoning may explain the variable distribution of *LCN2* (NGAL) beyond the collecting ducts and limbs of Henle in different kidney biopsies and mouse models (Figure 1). Whether the combination of chronic inflammatory disease with other insults leads to a molecular and cellular phenotype equivalent to ischemia or to a phenotype that represents a third, hybrid phenotype will be solved by kinetic and steady-state RNA analyses in the future.

In summary, Uprt RNA tagging is a powerful technique that provides time-resolved snapshots of RNA expression in a single cell type or kidney segment. The application of this technique to the study of acute failure of the kidney’s excretory function reveals a remarkably fine-tuned response: rather than a singular final common pathway (Figure 1), (*i*) a stimulus can activate different genes in different cells (Figures 1, 5, and 6), (*ii*) a different stimulus can induce different sets of genes, and (*iii*) the patterning of gene expression is transient (Figure 2E). A complete spatial and temporally resolved map should help develop approaches to precisely phenotype acute kidney diseases.

## Methods

### Animal models.

The cDNA of *Uprt* from *Toxoplasma gondii* (*T*. *gondii*) was inserted by LR Gateway conversion reaction into a Rosa26 targeting vector, *pBigT-DEST*. The final targeting vector was verified by sequencing and restriction digestion. *Uprt*-knockin mice were generated by standard homologous recombination in mouse embryonic stem (ES) cells (KV1 ES line, a 129B6N hybrid ES line, Columbia University Herbert Irving Comprehensive Cancer Center: Genetically Modified Mouse Models Shared Resource) and blastocyst injection of targeted ES cells into C57BL/6J blastocysts (mice from The Jackson Laboratory) to generate germline chimeras. The presence of transcriptional stop cassette *PGK-neo-pA-3xpA* prevented downstream *Uprt* cDNA expression, while mating with mice expressing tissue/cell-specific Cre activated the expression of *Uprt* cDNA in a cell-specific manner. Because mammalian cells do not have functional *Uprt*, cell-specific expression of *T*. *gondii Uprt* allowed incorporation of 4-TU (MilliporeSigma) in nascent mRNA in the Cre-targeted cells. To isolate the labeled mRNA, transcripts were thio-biotinylated in vitro and isolated on streptavidin beads (Miltenyi Biotec) for RNA-Seq. The *Rosa^Uprt/+^* mice were backcrossed with C57BL/6J for 5 generations and maintained as *Rosa^Uprt/Uprt^*. They were bred with Hoxb7Cre, Atp6v1b1Cre, and *Lrp2^CreERT2/+^* (see below) to generate *Hoxb7Cre Rosa^Uprt/+^*, *Atp6v1b1Cre Rosa^Uprt/+^*, and *Lrp2^CreERT2/+^ Rosa^Uprt/+^*, respectively.

We created *Lrp2^CreERT2/+^* by recombineering, replacing the translational STOP (TAG) with P2a-CreERT2-frt-neo-frt cassette. The modified bacteria artificial chromosome clone was confirmed by junctional PCR and sequenced across the P2a-CreERT2-frt-neo-frt cassette as well as the 5*′* and 3*′* of the insertion site. The *Lrp2^CreERT2/+^* mice were then generated by standard homologous recombination in mouse ES cells and blastocyst injection of targeted ES cells. The P2a sequence allows expression of *Lrp2* and *CreERT2* from the same allele. The Neo cassette was removed by crossing Actin-Flpe mice (The Jackson Laboratory), and the resulting *Lrp2^CreERT2/+^* mice were maintained as homozygotes (Supplemental Figure 5).

For RNA-Seq, both sexes were used at 5 months old, while 3-month-old mice were used for all other experiments. To ubiquitously label all cells in the mouse, we deleted the stop cassette by breeding the *Rosa^Uprt/Uprt^* mouse with the *EIIaCre* mouse (The Jackson Laboratory strain 003724) (89) and subsequently removed the EIIaCre to obtain a mouse line called *Rosa^UprtdSTOP/+^*. *Zfp36^–/–^* (90) (gift from Perry Blackshear, Department of Biochemistry, Duke University, Durham, North Carolina, USA), *Hoxb7Cre* (91) (The Jackson Laboratory strain 004692), *Atp6v1b1Cre* (92) (gift from Raoul Nelson, Division of Nephrology, Department of Pediatrics, University of Utah, Salt Lake City, Utah, USA), *Rosa^mTmG/mTmG^* (93) (The Jackson Laboratory strain 007676), and *Rosa^nT-nG/nT-nG^* (94) (The Jackson Laboratory strain 023035) mice were described previously. All primers for genotyping are listed in Supplemental Table 12.

For volume depletion, adult mice with known genotypes had restricted access to water and food for 24, 48, or 72 hours. For the ischemia/reperfusion injury model, adult mice were anesthetized with ketamine (100 mg/kg body weight) and xylazine (10 mg/kg body weight) and laparotomy was performed and both renal arteries were occluded with microvascular clamps for 10 minutes. During the procedure, the animals were warmed (36.5°C), and treated with 300 μL of sterile saline to compensate for volume losses.

### Validation of Cre drivers.

*Rosa^nT-nG^* 2-color fluorescent reporter mice were generated by breeding *Rosa^nT-nG^* mice with *Hoxb7Cre* or *Atp6v1b1Cre* mice to label collecting duct cells with GFP and untargeted kidney cells with red fluorescent protein (Tomato). Kidneys from *Hoxb7Cre Rosa^nT-nG/nT-nG^* reporter mice or *Atp6v1b1Cre Rosa^mTmG/mTmG^* reporter mice were harvested and digested using Multi Tissue Dissociation Kit 2 (Miltenyi Biotec). Quartered tissues were dissociated with Buffer X, Buffer Y, Enzyme P, Enzyme D, and Enzyme A using a MACS Dissociator (all from Miltenyi Biotec; 30 minutes at 37°C). Cells were collected by centrifugation (800*g* for 10 minutes), filtered (100- and 40-micron cell strainers from Falcon, Corning), and treated with live cell dye, Calcein Violet AM (0.5 μM, Thermo Fisher Scientific C34858). Live duct cells (Calcein AM^+^ and GFP^+^) were collected by BD FACSAria II Cell Sorter (Columbia University Flow Cytometry Core).

### Isolation of nascent RNA.

4-TU (MilliporeSigma 440736, 10 mg) was dissolved in DMSO (100 μL), diluted with shaking for 30 minutes in 50 mM Tris/HCl pH 8.8 (900 μL) at 50°C in the dark, and inoculated (430 mg/kg body weight; i.p.).

Nascent RNA was isolated by harvesting kidneys 4 hours after inoculation and performing 2 purification steps. Total RNA (~250–400 μg RNA per kidney) was first isolated from kidneys by homogenization (PowerGen125; 30 seconds; maximal output; Thermo Fisher Scientific) in TRIzol (4 mL; Life Technologies 15596-026). Next, 4-TU–labeled RNA was biotinylated with a sulfhydryl-specific biotin reagent (EZ-link Biotin HPDP; 1 mg/mL, Pierce Biotechnology, 21341) dissolved in *N,N*-dimethylformamide (MilliporeSigma D4551) at 37°C. Thio-biotinylation was initiated by mixing 100 μg of total RNA with the EZ-link Biotin HPDP reagent (final concentration: 0.2 mg/mL) in 1 mL of 10 M Tris/HCl at pH 7.5 with 1 mM EDTA (25°C). The reaction was terminated after 3 hours by removing free biotin with chloroform extraction (twice) and then solubilizing RNA in water (65°C; 10 minutes). Thio-biotinylated RNA was purified with streptavidin beads (100 μL, Miltenyi Biotec, 130-074-101) and magnetic columns (Miltenyi Biotec, 130-042-602). The beads were washed (100 mM Tris at pH 7.5, 10 mM EDTA, 1 M NaCl, 0.1% v/v Tween 20) at 65°C (3 washes) and at 25°C (3 washes), and thio-biotinylated RNA was then eluted with 5% (v/v) β-mercaptoethanol in 100 μL water. The RNA was concentrated with RNeasy MinElute Cleanup Kit (Qiagen, 74204) and stored in water (–80°C).

Concentrations of reagents were initially optimized by examining a range of Biotin HPDP (0, 50%, 100%, EZ-link) (Pierce Biotechnology, 21341) and a range of streptavidin beads (0, 50%, 100%, 200%), where 100% is the amount we currently utilize as per Gay and Doe (28). The capture of 4-TU–nascent RNA by the beads was measured by a Bioanalyzer (Supplemental Figure 2). The pulldown was optimal at 100% cross-linking reagent and 50% or 100% beads.

### RT-qPCR.

Biotinylated RNA purified from 200 μg total kidney RNA was subjected to DNase I treatment, followed by cDNA synthesis using random primers (Superscript III, Invitrogen). cDNA (2 ng) was used for quantitative PCR reactions (SYBR Select Master Mix, Thermo Fisher Scientific). Primers are listed in Supplemental Table 12.

### Human and mouse ISH.

ISH on FFPE human and mouse kidney tissues was performed using the chromogenic RNAscope 2.5 HD reagent kit (RED, ACD, catalog 322350) and the RNAscope 2.5 HD duplex reagent kit (ACD, catalog 322430) according to the manufacturer’s protocols. The following probes of interest were used in single or dual channel: Hs-LCN2 (catalog 559441; Supplemental Figure 1B without dilution) and Hs-LCN2-C2 (catalog 559441-C2; Figure 1, A and B, at 1:600; Supplemental Figure 1A at 1:3000), Hs-HAVCR1-O1 (catalog 538081; Figure 1B without dilution), Hs-AQP2 (catalog 434861; Figure 1, A and B, without dilution), Mm-Lcn2-C2 (catalog 313971-C2; Figure 1, C and D, at 1:1600 dilution), and Mm-Havcr1 (catalog 472551; Figure 1, C and D, at 1:25 dilution) (Supplemental Table 13). Briefly, tissue sections were deparaffinized and rehydrated with xylene and ethanol washes, blocked with hydrogen peroxidase, heated in target retrieval buffer, and digested by protease. The tissue sections were then incubated with RNAscope target probe(s) at 40°C in a hybridization oven for 2 hours. RNA signal was amplified using the kit’s amplification system conjugated to alkaline phosphatase and/or HRP, and signal was detected using chromogenic substrate solutions. Tissue sections were then counterstained with hematoxylin, dried in a 60°C oven, and mounted for image analysis. Bright-field images were captured using the Olympus IX73 inverted microscope under low (100×) and high (600×) magnifications.

### Dot blot.

Total RNA from *Rosa^Uprt/+^* and *EIIaCre Rosa^Uprt/+^* kidneys was thio-biotinylated in vitro, then spotted on zeta membranes. The thio-biotinylated RNAs were detected with streptavidin-HRP and visualized with ECL.

### Immunoblots.

Immunoblots were used to measure urinary Ngal with 4% to 15% sodium dodecylsulfate–polyacrylamide gel electrophoresis (Bio-Rad Laboratories) and detected using goat anti-mouse Ngal (1:1000; R&D Systems, catalog AF1857) and goat polyclonal HRP secondary antibodies (1:5000; Jackson ImmunoResearch, catalog 705-035-147) (Supplemental Table 13).

### Immunohistochemistry.

Mouse kidneys were fixed (4% paraformaldehyde/0.1 M phosphate buffer at 4°C overnight), transferred to 30% sucrose/0.1 M phosphate buffer (4°C overnight), and embedded in O.C.T. Compound (Tissue-Tek). Frozen sections of 20 μm were used for immunofluorescence staining with goat anti mouse Kim-1 (1:200; R&D Systems, catalog AF1817) and rabbit anti mouse Megalin (1:400; Abcam catalog ab76969) (Supplemental Table 13). Fluorescent secondary antibodies, Alexa Fluor 594–AffiniPure F(ab*′*)2 Fragment Donkey Anti-Goat and Alexa Fluor 488–AffiniPure F(ab*′*)2 Fragment IgG Donkey Anti-Rabbit IgG (1:1000; Jackson ImmunoResearch Laboratories), were used for Kim-1 and Megalin identification, respectively. All slides were costained with DAPI to identify nuclei.

### RNA-Seq.

RNA was quantified by Agilent 2100 Bioanalyzer with the RNA pico kit (Agilent 5067-1513). We submitted 300 ng of total RNA and 20–60 ng of purified biotinylated RNA to Columbia University Genome Center for poly(A) mRNA purification, library construction, and RNA-Seq (30 million reads; Illumina HiSeq 2000).

### Data analysis.

Illumina RTA was used to perform base calling, and CASAVA (version 1.8.2) was used for converting base call files (.BCL) to FASTQ format and for performing sequence adaptor trimming. Reads were then trimmed using Trimmomatic version 0.36 (LEADING: 10 TRAILING: 10 MINLEN:30) and then aligned to the mouse reference genome (mm10) using STAR aligner version 2.5.3a (default settings). Count tables were generated with HTSeq (version 0.6.1p2) using default settings. Gene expression levels were normalized to transcript length and library size (TPM). Transcripts with fewer than 2 counts across all samples were removed. Statistical analysis was performed in R (version 3.3.2), and additional Bioconductor packages were part of release 3.4. Data were deposited: Gene Expression Omnibus series GSE99084; https://www.ncbi.nlm.nih.gov/geo/query/acc.cgi?acc=GSE99084

### Identification of genes and pathways.

DEGs were identified using DESeq2 package (version 1.14.1) modeling for the sex, RNA pulldown, and treatment effects (design = ~sex + group, group represents the RNA and treatment). Pathway enrichment analysis was performed using our data set (padj < 0.01; log_2_ fold change ≥ 1) against KEGG (release 82.0), Reactome (version 60), and WikiPathways (version 20170510) databases. *P* values were calculated using hypergeometric distribution in R and corrected for multiple comparisons using the Benjamini-Hochberg procedure. The effects of batch and sex were displayed using principal component analysis (Supplemental Figure 4).

### Statistics.

All pairwise comparisons (e.g., transcript levels of genes between EIIaCre and HoxB7Cre, relative mRNA expression of genes in different cell types over time compared with day 0, and creatinine, BUN, and HCT for ischemia and volume depletion models compared with controls) were analyzed using 2-tailed Student’s *t* test. *P* values of less than 0.05 were considered statistically significant. All data are presented as mean ± SD.

### Study approval.

All mouse experiments were approved by the IACUC at Columbia University (protocol no. AC-AABG4552). This study was approved by the Institutional Review Board of Columbia University Irving Medical Center, New York, New York, USA (protocol no. AAAO2107).

## Author contributions

Order of co–first authors was assigned by the originator first, followed by data scientist and then experimentalist (mouse models). THS subcloned *Rosa Uprt* and *Lrp2^CreERT2^*, established the mouse models, and performed and analyzed most experiments with RNA extractions and RNA-Seq; KX, JS, ADL, CSL, SM, NP, MSG, EM, TG, NA, QYZ, FL, PIG, AB, and YL performed or analyzed mouse models of ischemia and volume depletion; JS, KX, JSS, AJ, SA, SKS, NPT, NP, and RB performed informatics and biomarker analyses; MC, KX, and VD evaluated human tissue and performed ISH; and KX, BDDO, KK, THS, and JB wrote the manuscript with the help of all the authors.

## Supplementary Material

Supplemental data

Supplemental table 1

Supplemental table 2

Supplemental table 3

Supplemental table 4

Supplemental table 5

Supplemental table 6

Supplemental table 7

Supplemental table 8

Supplemental table 9

Supplemental table 10

Supplemental table 11

Supplemental table 12

Supplemental table 13

## Version 1 03/01/2022

In-Press Preview

## Version 2 03/22/2022

Electronic publication
