# Supplementary material for: Snapshots of nascent RNA reveal cell- and stimulus-specific responses to acute kidney injury
Source: JCI Insight. 2022 Mar 22;7(6):e146374. doi: 10.1172/jci.insight.146374 (PMC8986083; doi:10.1172/jci.insight.146374)
Supplement: Supplemental data [file jciinsight-7-146374-s065.pdf]

Snapshots of Nascent RNA Reveal Cell- and Stimulus-Specific Responses to Acute Kidney Injury

Tian Huai Shen*[1], Jacob Stauber*[1], Katherine Xu*[1], Alexandra Jacunski[2], Neal Paragas[3], Miriam Callahan[1], Run Banlengchit[1], Abraham Levitman[1], Beatriz Desanti De Oliveira[1], Andrew Beenken[1], Madeleine S. Grau[1], Edwin Mathieu[1], Qing-Yin Zhang[1], Yuanji Li[1], Tejashree Gopal[1], Nathaniel Askanase[1], Siddarth Arumugam[4], Sumit Mohan[1,5], Pamela Good[6], Jacob Stevens[1], Fangming Lin[6], Samuel K. Sia[4], Chyuan-Sheng Lin[7], Vivette D'Agati[7], Krzysztof Kiryluk[1], Nicholas P. Tatonetti†[2] and Jonathan Barasch†[1]

[1]Department of Medicine, Columbia University, New York, NY

[2]Department of Biomedical Informatics, Columbia University, New York, NY

[3]Department of Medicine, University of Washington, Seattle, WA

[4]Department of Biomedical Engineering, Columbia University, New York, NY

[5]Department of Epidemiology, Columbia University, New York, NY

[6]Department of Pediatrics, Columbia University, New York, NY

[7]Department of Pathology and Cell Biology, Columbia University, New York, NY

Supplemental Figures and Figure Legends

*Equal Contribution

† Corresponding Authors
†Nicholas Tatonetti PhD
Department of Biomedical Informatics
Columbia University
Presbyterian Hospital PH20-402
630 West 168th St
New York, NY 10032
npt2105@columbia.edu

†Jonathan Barasch MD PhD
Department of Medicine and Pathology and Cell Biology
Columbia University
1150 St Nicholas Ave,
New York, NY 10032
jmb4@columbia.edu

Key Words: Kidney, Serum Creatinine, Acute Tubular Injury, Acute Kidney Injury, Volume Depletion, Uracil Phosphoribosyl Transferase, TU-Tagging.

Figure S1. **Patterning of Gene Expression.** (A) Human Kidney Biopsy. Nephrectomy- 18 minutes of warm ischemia followed by cold ischemia. *LCN2* (NGAL) is expressed in large tubular ducts in excess of surrounding proximal tubules. RNAscope Red-NGAL probe (Cat. No. 559441-C2) was diluted 1:3000 (5 times the dilution used in Figure 1). (B) Acute tubular injury in the setting of pyelonephritis. *LCN2* (NGAL) is expressed in tubules. RNAscope Green-NGAL probe (Cat. No. 559441). Bar = 20μm.

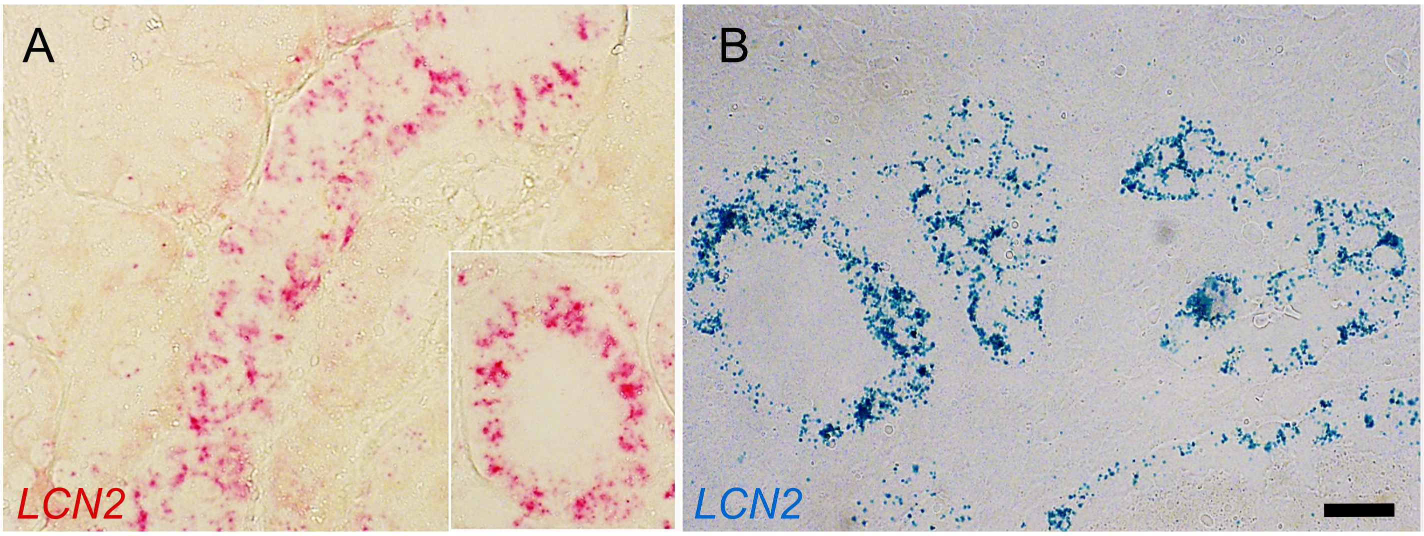

Figure S1

Figure S2. **Optimization of the pulldown of 4-TU labeled RNA.** Total RNA extracts from kidneys of 3 month old mice *EIIACre; Rosa*$^{Uprtf/+}$ were thio-biotinylated and thio-RNA recovered using avidin-bead precipitation. A range of Biotin HPDP (0%, 50%, 100%, Cx-L), and a range of avidin beads (0, 50%, 100%, 200%) where evaluated by BioAnalyzer detection of RNA. Note that the pulldown was optimal at 100% crosslinking reagent and 50% or 100% beads. 100% refers to recommendations of Gay et al (28).

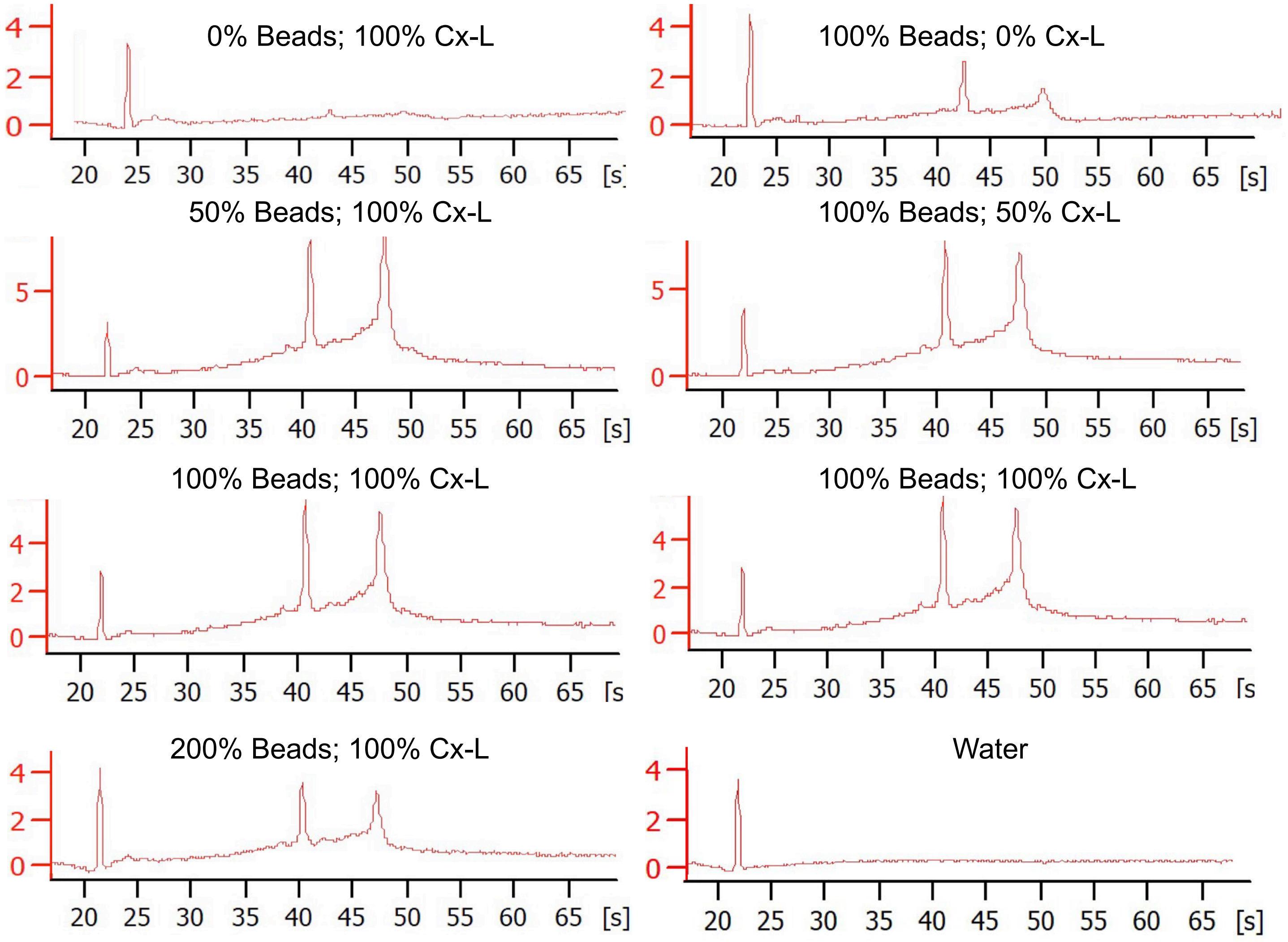
0% Beads; 100% Cx-L
100% Beads; 0% Cx-L
50% Beads; 100% Cx-L
100% Beads; 50% Cx-L
100% Beads; 100% Cx-L
100% Beads; 100% Cx-L
200% Beads; 100% Cx-L
Water
[s]

Figure S2

Figure S3. **Validation of Cell Specific Cre Driven RNA Isolation.** *Hoxb7Cre;Rosa*$^{nT\text{-}nG/nT\text{-}nG}$ and *Atp6v1b1Cre;Rosa*$^{mTmG/mTmG}$ mice revealed collecting duct specific GFP staining. The GFP^{+} cells were isolated from 3 month old *Hoxb7Cre;Rosa*$^{nT\text{-}nG/nT\text{-}nG}$ and *Atp6v1b1Cre;Rosa*$^{mTmG/mTmG}$ mice by FACS (gated for Calcein AM^{+} live cells) from dissociated kidneys and then subjected to RT-qPCR. Note that Hoxb7Cre enriches for PC genes while Atp6v1b1Cre enriches for IC genes, despite overlapping Cre domains. Scale Bar =100μm.

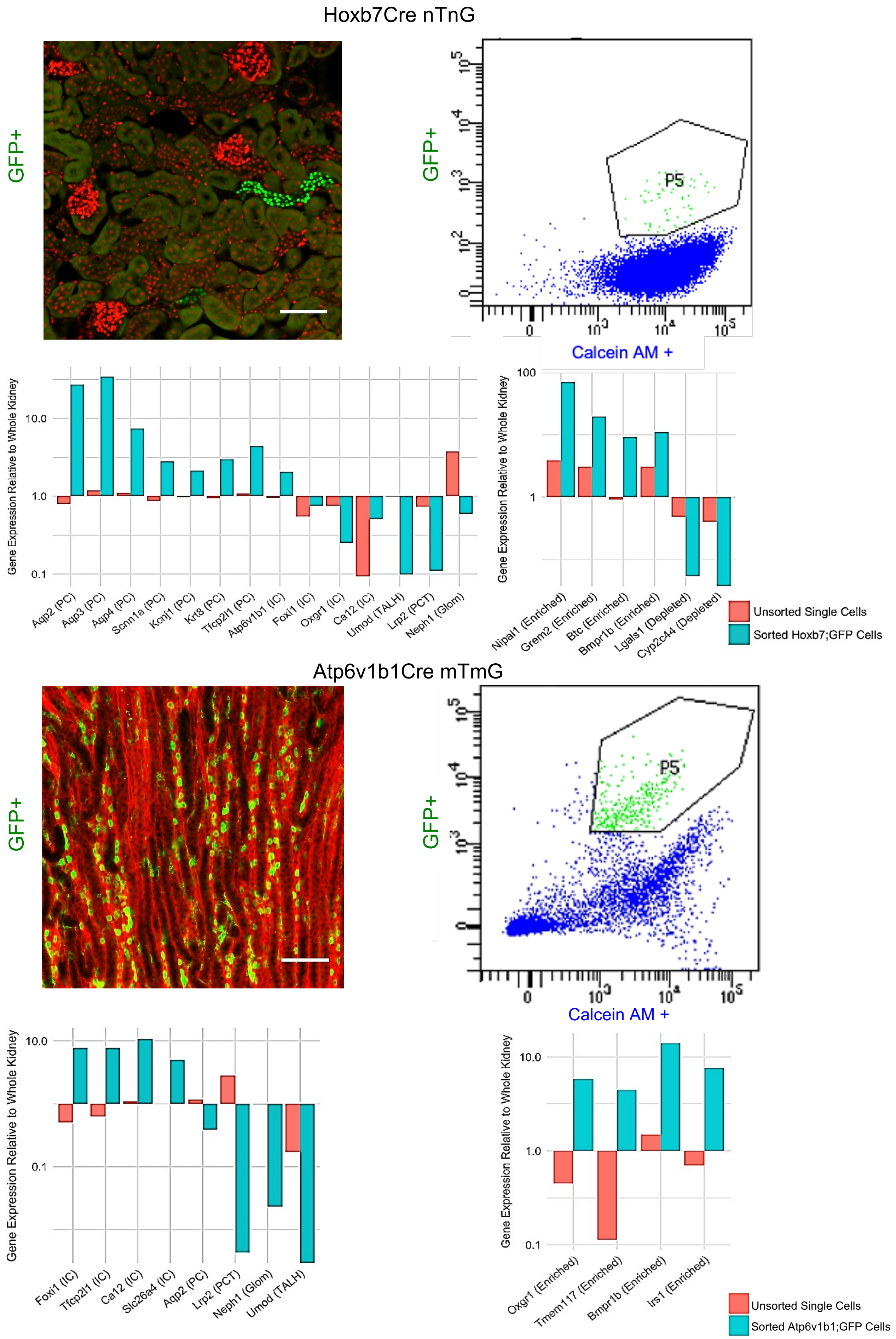
Hoxb7Cre nTnG
GFP+
GFP+
P5
Calcein AM +
Gene Expression Relative to Whole Kidney
10.0
1.0
0.1
Aqp2 (PC)
Aqp3 (PC)
Aqp4 (PC)
Scnn1a (PC)
Kcnj1 (PC)
Krt8 (PC)
Tfcp2l1 (PC)
Atp6v1b1 (IC)
Foxi1 (IC)
Oxgr1 (IC)
Ca12 (IC)
Umod (TALH)
Lrp2 (PCT)
Neph1 (Glom)
Gene Expression Relative to Whole Kidney
100
1
Nipal1 (Enriched)
Grem2 (Enriched)
Btc (Enriched)
Bmpr1b (Enriched)
Lgals1 (Depleted)
Cyp2c44 (Depleted)
Unsorted Single Cells
Sorted Hoxb7;GFP Cells
Atp6v1b1Cre mTmG
GFP+
GFP+
P5
Calcein AM +
Gene Expression Relative to Whole Kidney
10.0
0.1
Foxi1 (IC)
Tfcp2l1 (IC)
Ca12 (IC)
Slc26a4 (IC)
Aqp2 (PC)
Lrp2 (PCT)
Neph1 (Glom)
Umod (TALH)
Gene Expression Relative to Whole Kidney
10.0
1.0
0.1
Oxgr1 (Enriched)
Tmem117 (Enriched)
Bmpr1b (Enriched)
Irs1 (Enriched)
Unsorted Single Cells
Sorted Atp6v1b1;GFP Cells

Figure S3

Figure S4. **Principal Component Analysis.** (**A**) Inspection of variance across PC1 and PC2 revealed that PC1 distinguished total kidney RNA vs *Hoxb7Cre;Rosa*$^{Uprt/+}$ and *Atp6v1b1Cre;Rosa*$^{Uprt/+}$ pulldowns while PC2 distinguished male from female mice at baseline. (**B**) Inspection of variance across PC1 and PC2 revealed that PC1 distinguished volume depletion from ischemia and PC2 distinguished *Hoxb7Cre;Rosa*$^{Uprt/+}$ and *Atp6v1b1Cre;Rosa*$^{Uprt/+}$ pulldowns. Volume Depleted mice, n=7; Ischemic mice, n=7; Control mice, n=8.

A

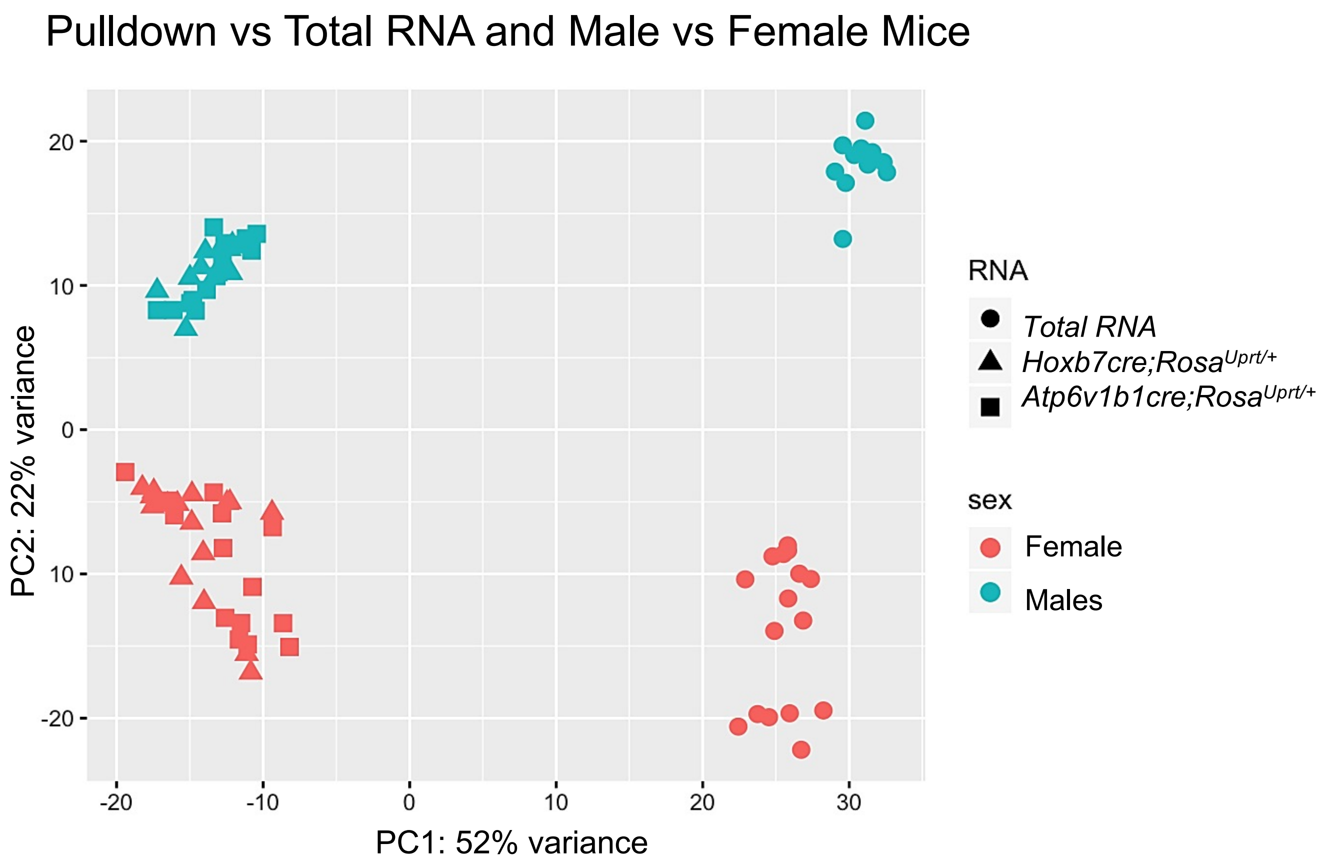

Pulldown vs Total RNA and Male vs Female Mice
PC2: 22% variance
PC1: 52% variance
RNA
Total RNA
Hoxb7cre;Rosa^Uprt/+
Atp6v1b1cre;Rosa^Uprt/+
sex
Female
Males

B

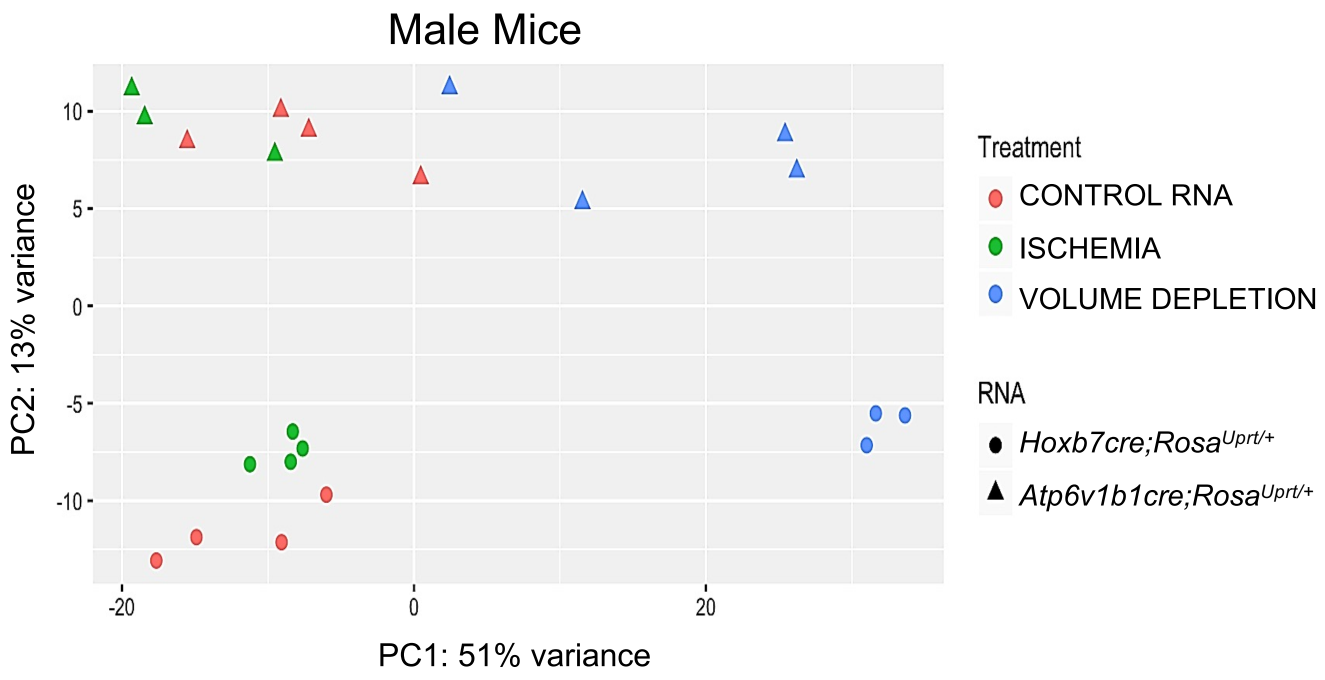

Male Mice
PC2: 13% variance
PC1: 51% variance
Treatment
CONTROL RNA
ISCHEMIA
VOLUME DEPLETION
RNA
Hoxb7cre;Rosa^Uprt/+
Atp6v1b1cre;Rosa^Uprt/+

Figure S4

Figure S5. **Generation of *Lrp2*$^{CreERT2/+}$**: Lrp2 translational STOP (TAG) was replaced with the "P2a-CreERT2-frt-neo-frt" cassette, permitting the expression of both *Lrp2* and *CreERT2* from the same allele. The Neo cassette was removed by crossing Actin-Flpe mice.

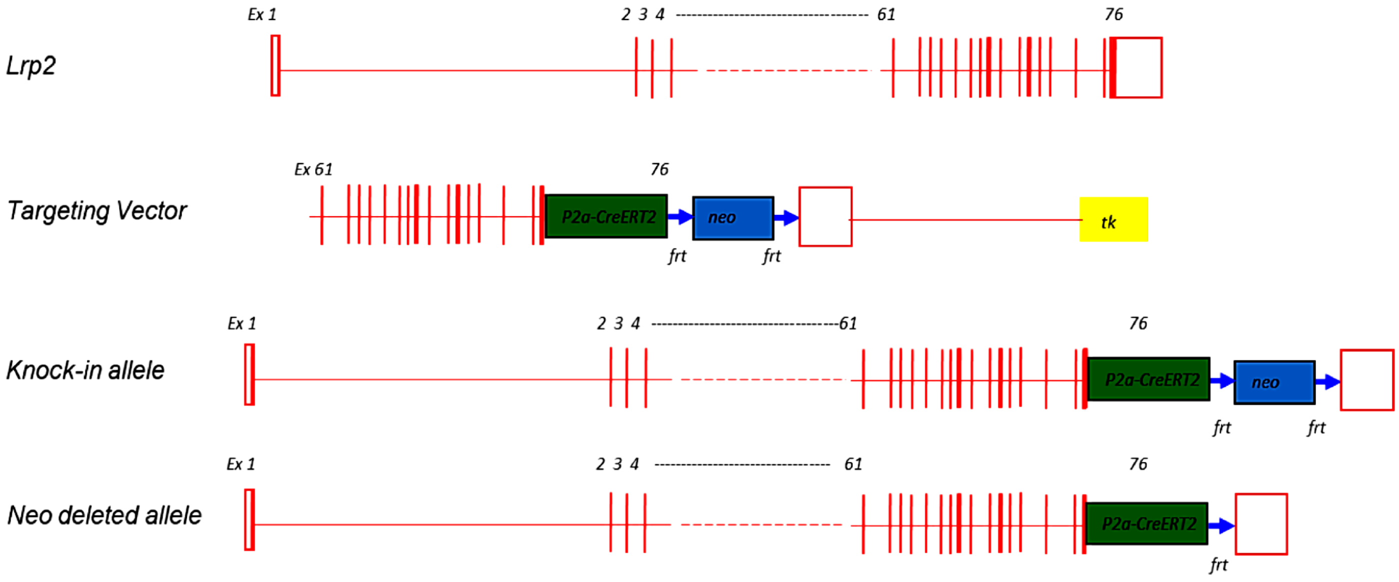
Ex 1
2 3 4
61
76
Lrp2
Ex 61
76
Targeting Vector
P2a-CreERT2
neo
frt
frt
tk
Ex 1
2 3 4
61
76
Knock-in allele
P2a-CreERT2
neo
frt
frt
Ex 1
2 3 4
61
76
Neo deleted allele
P2a-CreERT2
frt

Figure S5

Figure S6. **Time-dependent gene expression in mouse model of volume depletion**. GFP labeled intercalated cells were isolated by FACS from *Atp6v1b1Cre;Rosa*$^{mTmG/mTmG}$ 3 month old mice subject to volume depletion for 0, 1, 2, and 3 days and assayed by RT-qPCR using *Actb* as reference. mRNA expression at day 0 (control) was set as 1. The asterisks indicate Bonferroni adjusted significance: *p<0.05, **p<0.01, ***p<0.001, respectively compared to day 0 control (two tailed t test); ns indicates no significance. n=8. Data should be compared to Figure 2E.

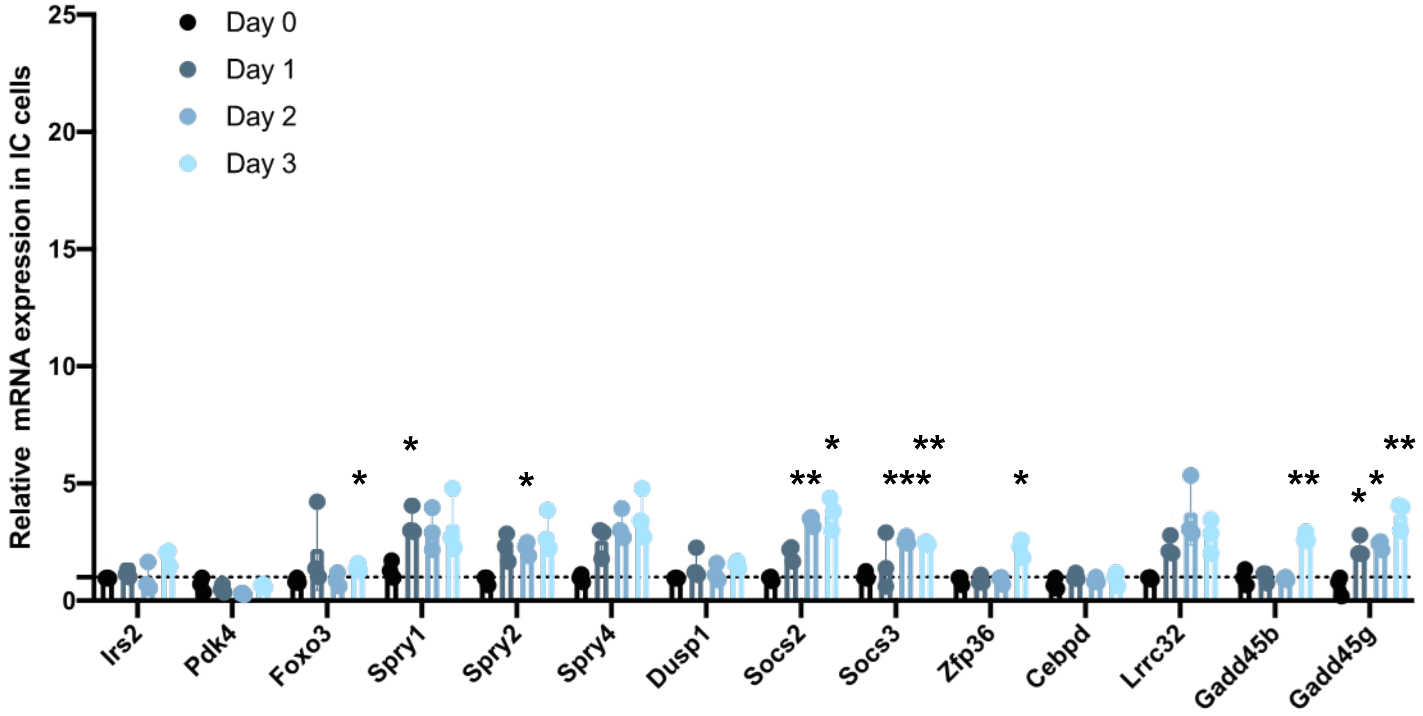

Relative mRNA expression in IC cells
0
5
10
15
20
25
Day 0
Day 1
Day 2
Day 3
Irs2
Pdk4
Foxo3
Spry1
Spry2
Spry4
Dusp1
Socs2
Socs3
Zfp36
Cebpd
Lrrc32
Gadd45b
Gadd45g

Figure S6

Figure S7. **Strengths and weaknesses of different transcriptomic methods.**

Method	Strengths	Weaknesses
UPRT Labeling<br><br>Sequencing Nascent RNA	Labels RNA in a specific cell type within intact tissue, preserving cellular interactions and organ physiology.<br><br>Labels kinetically active nascent transcription at a chosen time point<br><br>Labels several types of RNA: mRNA, noncoding RNA, miRNA, and rRNA.<br><br>Isolates adequate amounts of nascent RNA even derived from rare or dispersed cells without batch pooling.<br><br>Isolates nascent RNA without organ dissection or tissue dissociation.<br><br>No effect on mouse viability. No variation in the expression of UPRT.	Depends on Cre drivers. Useful only in species that can express Cre.<br><br>UPRT measures nascent transcripts which do not fully map to steady state transcriptomic databases.<br><br>May over-represent uracil-rich RNA, impacting pulldown efficiency and hence quantitative comparisons across all members of a gene-set.
Fluorescence activated cell sorting (FACS)<br><br>Sequencing Steady State RNA	Isolates different cell types on the basis of surface markers, gene expression, cellular size and granularity.<br><br>Isolates a quantifiable number of cells from a specific population (ranging from a single cell to millions of cells).<br><br>Isolates cells for analysis of steady state RNA by RNA-seq.	Tissue dissociation into a single cell suspensions results in selective cell isolation, cell stress and death.<br><br>Flow cytometry contributes to cell stress and cell death, yet requires living cells for successful sorting.<br><br>Cell recovery can vary 50-70%, potentially limiting rare cell types.<br><br>Depends on the availability of antibodies targeted to the cell surface antigens.
Single cell scRNA-seq<br><br>Sequencing Steady State RNA	Provides gene expression at single cell resolution<br><br>Categorizes heterogeneous cell types independently of predefined markers or assumptions regarding cell hierarchies<br><br>Able to discover novel cell types<br><br>Enables bioinformatic reconstruction of a dynamic cellular process such as development, differentiation, cell states and disease progression.	Depends on RNA preservation, including freezing protocols<br><br>Depends on tissue dissection or FACS purification to enrich for a specific cell types.<br><br>Tissue dissociation into single cell suspensions results in selective cell isolation and contributes to cell stress and cell death, yet living cells are required for successful analysis.<br><br>Low RNA capture efficiency, low sequencing coverage, low detection of specific transcripts, high dropout rates, high level of technical noise, and complex informatics<br><br>Contamination by dying or dead cells or by mixed multiple cells (i.e. doublets)

Figure S7

Figure S8. **Two Hits: Volume Depletion and Chronic Inflammation (Zfp36 KO)**. (**A**) *Zfp36* expression in *Zfp36*$^{+/+}$ *vs Zfp36*$^{-/-}$ 3 month mice *p<0.05; ***p<0.001 (**B**) *Pappa2* expression marked volume depletion in both wild type and *Zfp36*$^{-/-}$ mice. ***p<0.001; WT Control *vs* WT volume depletion; *Zfp36* knockout control *vs Zfp36* knockout volume depletion. Significance was determined by Student's two tailed t-test. Data represent mean ± SD. (**C**) Urine immunoblot for Ngal reveals limited expression of Ngal in wild type mice even after 72 hours of volume depletion. In contrast, volume depletion stimulated Ngal expression in mice with chronic inflammatory disease due to *Zfp36* deletion (3 month old littermates). Cloned purified Ngal proteins are standards. (**D**) Induction of Kim-1 in proximal tubules of *Zfp36*$^{-/-}$ mice exposed to volume depletion. In contrast, wild type control mice, *Zfp36*$^{-/-}$ control mice, and wild type (all 3 month old littermates) volume depleted mice all failed to express this marker of ischemic nephropathy. sCr was elevated equivalently in wild type and *Zfp36*$^{-/-}$ volume depletion; n=8 mice; WT Control *vs* WT volume depletion: p= 0.02; *Zfp36* knockout control *vs Zfp36* knockout volume depletion: p= 0.025. Bars= 25μM.

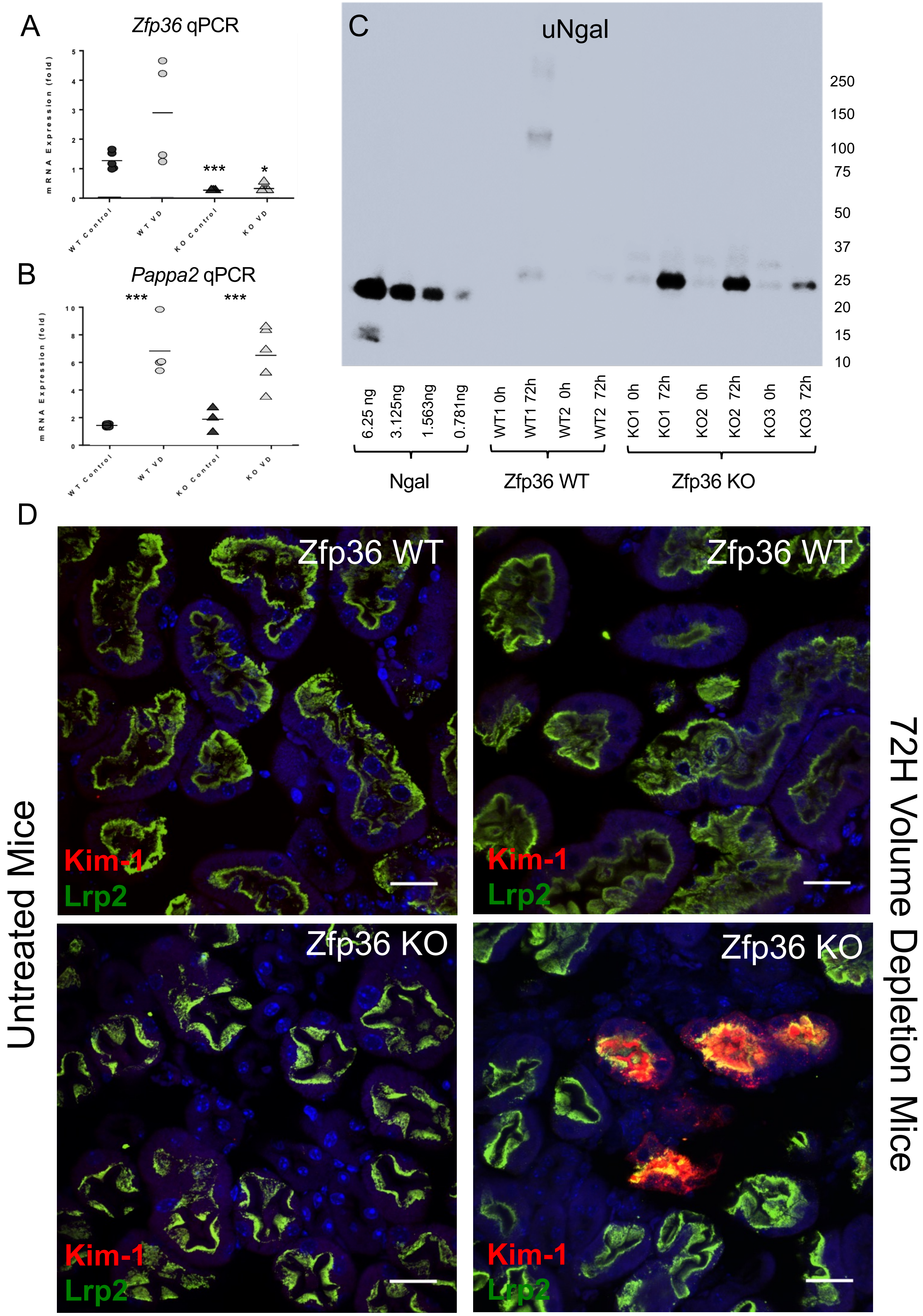
A
Zfp36 qPCR
mRNA Expression (fold)
WT control
WT VD
KO control
KO VD
***
*
B
Pappa2 qPCR
***
***
C
uNgal
250
150
100
75
50
37
25
20
15
10
6.25 ng
3.125ng
1.563ng
0.781ng
WT1 0h
WT1 72h
WT2 0h
WT2 72h
KO1 0h
KO1 72h
KO2 0h
KO2 72h
KO3 0h
KO3 72h
Ngal
Zfp36 WT
Zfp36 KO
D
Untreated Mice
72H Volume Depletion Mice
Zfp36 WT
Zfp36 KO
Kim-1
Lrp2

Figure S8

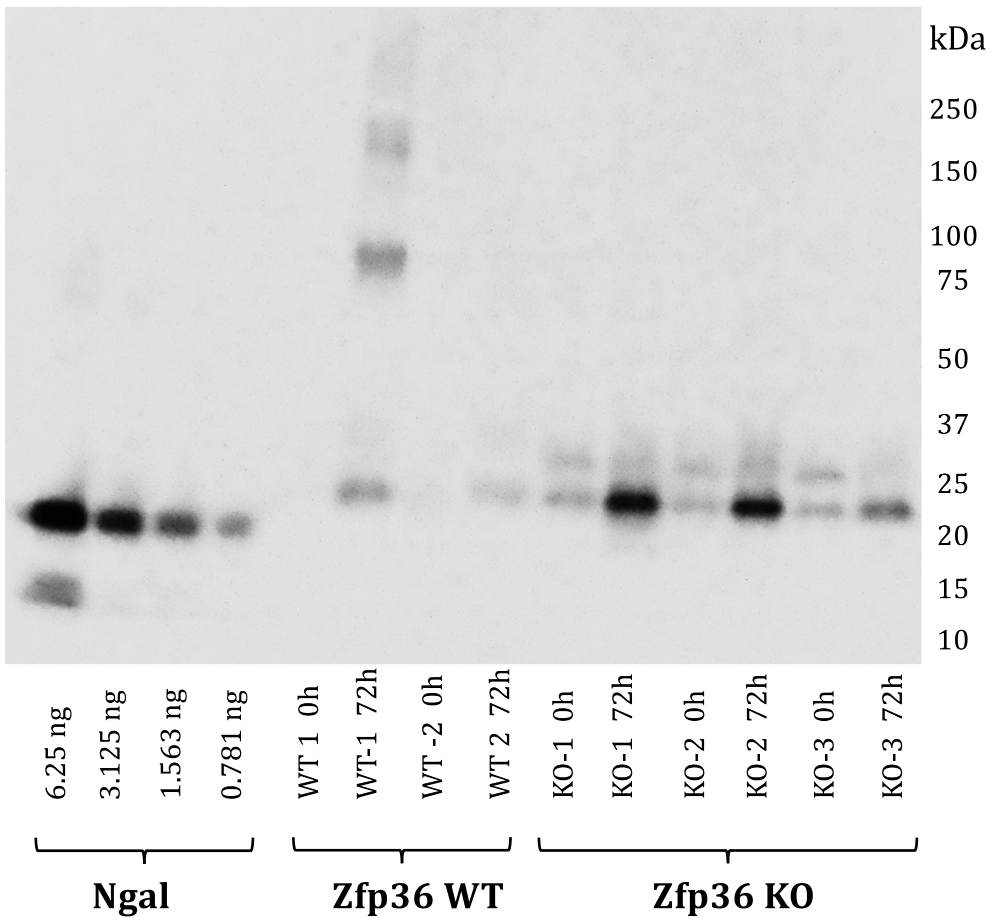

kDa
250
150
100
75
50
37
25
20
15
10
6.25 ng
3.125 ng
1.563 ng
0.781 ng
WT 1 0h
WT-1 72h
WT -2 0h
WT 2 72h
KO-1 0h
KO-1 72h
KO-2 0h
KO-2 72h
KO-3 0h
KO-3 72h
Ngal
Zfp36 WT
Zfp36 KO

Figure S9

Supplemental Tables: **Control, Ischemic and Volume Depleted Mice Datasets.**

Supplemental Table 1. Hoxb7Cre pulldown vs Total RNA values from male and female controls; p<0.05; n=21 mice

Supplemental Table 2. Atp6v1b1Cre pulldown vs Total RNA values from male and female controls; p<0.05; n=21 mice.

Supplemental Table 3. Hoxb7Cre pulldown ischemia vs Hoxb7Cre pulldown from control kidneys; p<0.05; male mice; n=7.

Supplemental Table 4. Hoxb7Cre pulldown volume depleted vs Hoxb7Cre pulldown from control kidneys; p<0.05; male mice; n=7.

Supplemental Table 5. Hoxb7Cre pulldown from ischemic vs volume depleted kidneys; p<0.05; log2 fold >1

Supplemental Table 6. Atp6v1b1Cre pulldown ischemia vs Atp6v1b1Cre pulldown from control kidneys; male; p<0.05; n=7.

Supplemental Table 7. Atp6v1b1Cre pulldown volume depletion vs Atp6v1b1Cre pulldown from control kidneys; p<0.05; male; n=8.

Supplemental Table 8. Atp6v1b1Cre pulldown from ischemic vs volume depleted kidneys; p<0.05; log2 fold >1

Supplemental Table 9. Atp6v1b1Cre vs Hoxb7Cre pulldowns from ischemia kidneys; p<0.05; log2 fold >1

Supplemental Table 10. Atp6v1b1Cre vs Hoxb7Cre pulldowns from volume depleted kidneys. p<0.05; log2 fold >1

Supplemental Table 11. Genes coding for secreted proteins comparing volume depletion and ischemic kidneys; p<0.05; log2 difference >1.

Supplemental Table 12. Primers

Supplemental Table 13. Probes and Antibodies
